# Synthesis and Evaluation of Powerful Antioxidant Dendrimers Derived from D-Mannitol and Syringaldehyde

**DOI:** 10.3390/ijms262210966

**Published:** 2025-11-12

**Authors:** Blessed Agbemade, Amanda R. Clark, Cyprien N. Nanah, Fati Haruna, Aundrea E. Stengard, Skylar A. Medes, Ashlyn M. Lapratt, Samara L. Morehouse, Rebecca L. Uzarski, Choon Young Lee

**Affiliations:** 1 Department of Chemistry and Biochemistry, Central Michigan University, Mount Pleasant, MI 48859, USA; agbem1b@cmich.edu (B.A.); clark3ar@cmich.edu (A.R.C.); nanah1cn@cmich.edu (C.N.N.); harun1f@cmich.edu (F.H.); steng1ae@cmich.edu (A.E.S.); medes1sa@cmich.edu (S.A.M.); lapra1am@cmich.edu (A.M.L.); moreh1sl@cmich.edu (S.L.M.); 2 Science of Advanced Materials Program, Central Michigan University, Mount Pleasant, MI 48859, USA; 3 Department of Biology, Central Michigan University, Mount Pleasant, MI 48859, USA; uzars2rl@cmich.edu

**Keywords:** antioxidant dendrimers, D-mannitol, syringaldehyde, DPPH assay, β-carotene bleaching, cell viability

## Abstract

Antioxidants play a crucial role in preventing oxidative damage and are therefore integral to various sectors, including healthcare, food preservation, cosmetics, and industrial applications. Their capacity to enhance overall health and improve the quality and shelf life of products in these domains underscores their significance. Two powerful antioxidant dendrimers were synthesized using D-mannitol as the core and syringaldehyde as the antioxidant-producing phenolic unit. The generation 1 (G1) dendrimer features 12 syringic units on its surface, while the generation 2 (G2) dendrimer has 24. Antioxidant capacities were assessed using the 2,2-diphenyl-1-picrylhydrazyl (DPPH) and the β-carotene bleaching assays. Based on IC50 values, the G2 (0.7 μM) and G1 (1.36 μM) dendrimers show 371- and 191-fold higher antioxidant activity, respectively, compared to the starting compound, syringaldehyde (260 μM). They are also 1257- and 647-times more effective than butylated hydroxytoluene (BHT) (880 μM). Overall, G2 is twice as potent as G1. The dendrimers also provide strong protection against β-carotene bleaching. At concentrations between 3.75 and 60 μM, G2 preserves 75% to 88% of β-carotene after 16 h at 45 °C, while G1 maintains 51% to 84%. In comparison, syringaldehyde and BHT provide significantly less protection, with ranges of 21% to 47% and 22% to 36%, respectively. Their greatly enhanced antioxidant capabilities are due to the numerous free-radical-scavenging sites created by phenolic hydroxyl groups, which have electron-donating groups at the *ortho* and *para* positions. In cell viability assays using macrophages, G1 caused a decrease in cell viability at ≥31 µM. Conversely, G2 exhibited a gradual reduction in cell viability across the concentration range of 0.1 µM to 111 µM, with viability declining from 11.1% to 96.3%, indicating that the larger G2 is more cytotoxic than the smaller G1.

## 1. Introduction

The unchecked activity of free radicals poses serious risks to human health, as these highly reactive species can lead to cellular damage and oxidative stress, which are linked to an elevated risk of devastating diseases such as cancer and cardiovascular conditions [1,2]. Moreover, their impact extends beyond health-related issues, as free radicals can compromise the integrity of various products, including food [3,4] cosmetics [5], and industrial materials like plastics [6]. Consequently, it is essential to implement robust measures to manage and mitigate oxidative damage caused by free radicals.

Antioxidants are compounds that can halt chain reactions triggered by radicals, thereby preventing oxidative damage. Both naturally occurring and synthetic antioxidants have been shown to decrease the rate of oxidative deterioration [7,8,9,10]. The ability of antioxidants to scavenge radicals in highly diverse settings makes the pursuit of antioxidants with different physicochemical structures and properties valuable. Therefore, significant initiatives are being pursued to develop a wide range of highly effective antioxidants [11,12,13].

Macromolecular antioxidants have been previously reported. They were usually formed by attaching antioxidants to a polymer [14,15,16,17] or part of a dendrimer surface [18,19] or encapsulating antioxidants within the internal spaces of dendrimers [20,21,22]. One of the main reasons for designing large antioxidants is to include multiple radical scavenging active sites that can enable greater free radical scavenging capacity [23]. Additionally, many large antioxidants can serve as nanocarriers for the co-delivery of small molecule antioxidants or drugs, enabling synergistic or complementary therapeutic effects [24]. By adjusting their size and surface chemistry, large antioxidants can be engineered to target specific tissues and reduce rapid metabolic degradation, leading to prolonged in vivo antioxidant activity [24].

The concept of antioxidant dendrimers, which have multiple antioxidant units on their surface and act as antioxidants on their own, is relatively recent. This contrasts with earlier approaches in which antioxidants were either encapsulated inside dendrimers, attached to part of the dendrimer surface, or conjugated to polymers. The attributes of dendritic antioxidants, such as surface chemistry, size, degree of branching, and number of surface groups, can be more easily modified to achieve specific and targeted properties compared to other large antioxidants. In addition, their well-defined structures can provide more consistent antioxidant properties and minimize batch-to-batch variations. However, there are only a few studies reporting dendrimers that function as antioxidants independently [25,26].

Our research focuses on designing and synthesizing highly effective antioxidants in dendritic structures that utilize structural features beneficial for antioxidant activities [27,28,29]. These features include multiple phenolic hydroxyl (OH) groups and electron-donating substituents positioned *ortho* and/or *para* to the OH groups [30,31,32,33]. Our previous studies have demonstrated that antioxidants in dendritic structures exhibit strong radical-scavenging activities. As the number of surface phenolic units increased, their potency also increased. However, the antioxidant dendrimers were all generation 1 and built using different scaffolds [27,28,29]. A direct comparison between antioxidants built on the same scaffolds is lacking. The current study aimed to investigate the hypothesis that the greater the number of antioxidant units, the stronger their antioxidant effects, using the same family of dendrimers.

We developed macromolecular dendrimers derived from syringaldehyde, incorporating a large number of syringic units in this study. This report details the synthesis of dendritic antioxidants containing either 12 or 24 syringic units, highlighting advancements that aim to enhance the mitigation of oxidative damage across various applications.

## 2. Results

### 2.1. Chemistry

**Scheme 1:**
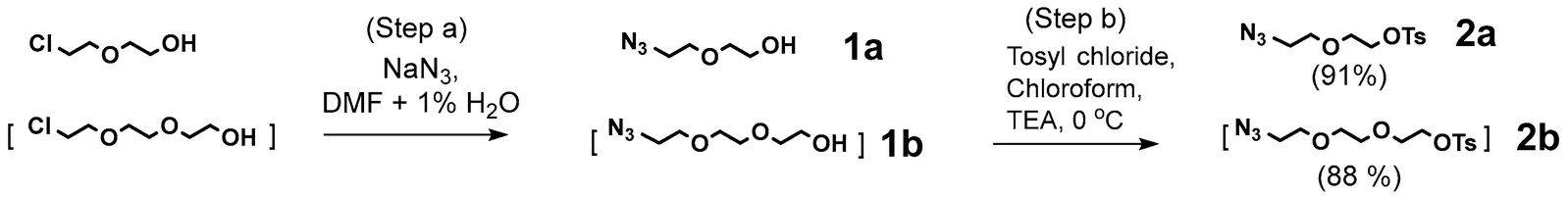
Synthesis of linkers.

**Scheme 2:**
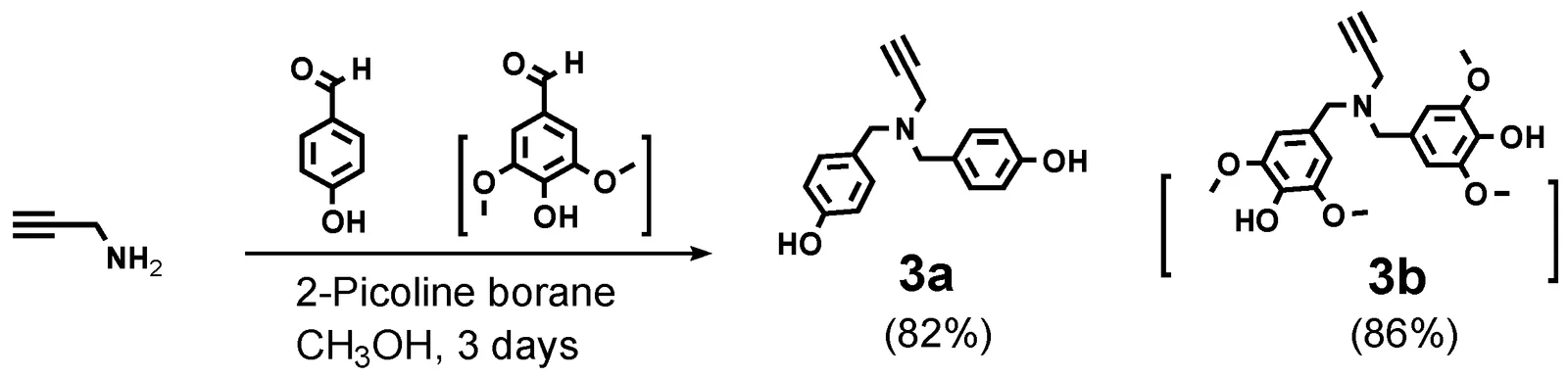
Synthesis of building blocks.

The target dendrimers were synthesized using a divergent approach, in which the process starts with a core and sequentially adds layers outward, creating a dendrimer that grows with each step. For the synthesis of target dendrimers, two linkers (Scheme 1) and two BBs (Scheme 2) were first prepared. The linkers were synthesized by converting 2-(2-chloroethoxy)ethanol and 2-(2-(2-chloroethoxy)ethoxy)ethanol into their azido derivatives, **1a** and **1b**, respectively, using sodium azide in DMF with 1% water [20]. Their OH groups were then tosylated using p-toluenesulfonyl chloride and triethylamine to produce **2a** (short linker) and **2b** (long linker), respectively (spectral data are shown in Supplementary Figures S1–S3 in Supplementary Materials).

Two building blocks (BBs) were synthesized. An internal BB (**3a**) was synthesized by reacting propargyl amine with 4-hydroxybenzaldehyde, using 2-picoline borane as the reducing agent (Supplementary Figures S4–S6). A surface BB (**3b**), which carries two antioxidant units, was also produced using the same method, by reacting propargylamine with syringaldehyde (4-hydroxy-3,5-dimethoxybenzaldehyde) (Supplementary Figures S7–S9).

**Scheme 3:**
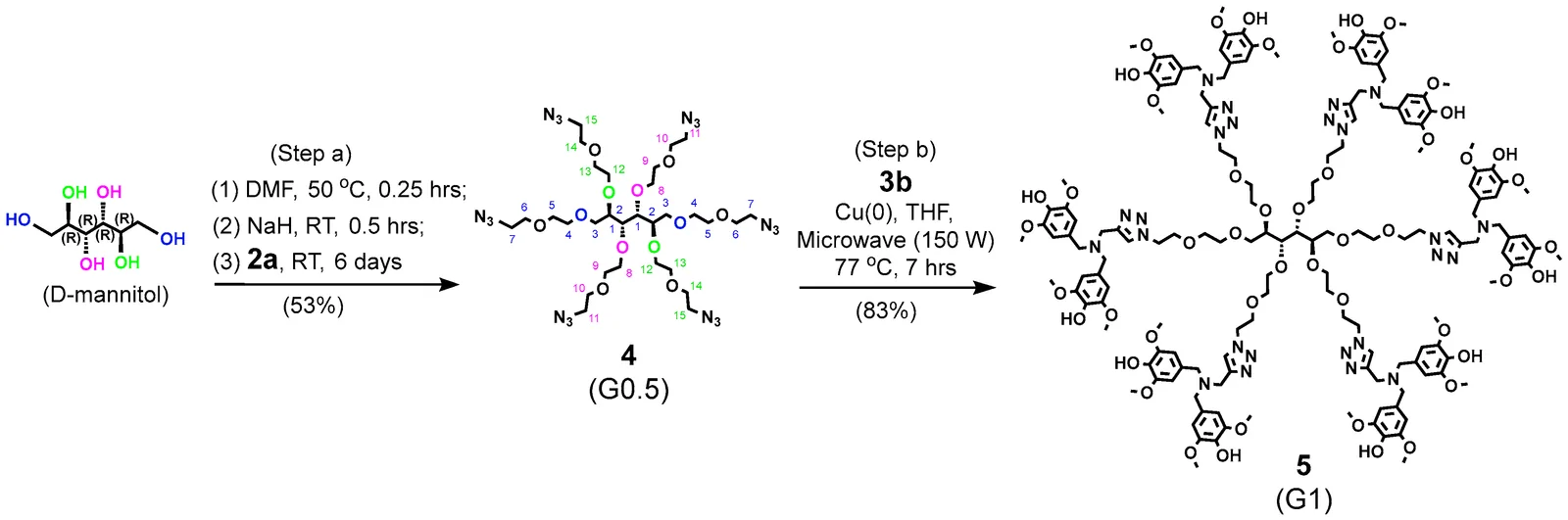
Synthesis of the G1 target dendrimer. Note: the same numbers with the same color across the two chains show identical chemical shifts in the NMR spectra.

To construct dendrimers, D-mannitol, a six-carbon chain with each carbon atom bonded to an OH group, served as the core (Scheme 3). Its four internal OH groups have R configurations, causing all six OH groups to orient in different directions. This arrangement helps reduce steric hindrance in subsequent reactions involving these groups.

As shown in Scheme 3 (step a), D-mannitol was derivatized with compound **2a** to produce the Generation 0.5 (G0.5) dendrimer (compound **4**). The reaction was performed in anhydrous DMF using sodium hydride (NaH). D-mannitol is highly soluble in water but only slightly soluble in DMF. To enhance solubility in DMF, the suspension was heated to 50 °C for 15 min and then cooled to room temperature before adding NaH. The powdered NaH was added directly to the reaction quickly, without pre-weighing. Once the balloon attached to the septum began to expand, signaling deprotonation, six equivalents of neat compound **2a** were added via a cannula needle. The reaction required 5–6 days. As more linkers attach to the OH groups of D-mannitol, which has six consecutively arranged OH groups on a six-carbon chain, the reaction rate slows down due to increased steric hindrance from the already attached linkers. Although other bases, such as DBU and DIPEA, were also tested, they did not improve the reaction rate; instead, they reacted with the linker, converting it to its alkene form. Analysis of compound **4** was performed using LC/MS (ESI-TOF) and NMR. Compound **4** was detected as [M + H]^+^, [M + NH_4_]^+^, [M + Na]^+^, and [M + K]^+^ ions, exhibiting monoisotopic masses of 861.4431, 878.4692, 883.4243, and 899.3969 amu, respectively. The theoretical monoisotopic masses for these adducts are 861.4398, 878.4663, 883.4217, and 899.3957 amu, respectively. (Supplementary Figure S10). Compound **4** was characterized using ^1^H and ^13^C NMR (Supplementary Figures S11 and S12). The correlation 2D NMR techniques, such as ^1^H–^1^H COSY, ^1^H–^13^C HSQC, and ^1^H–^13^C HMBC, were also employed to accurately assign the signals and determine H and C connectivity (HSQC is shown in Supplementary Figure S13). Our assignments were compared with the chemical shifts and integration values estimated by a program (MestreNova NMRPredict version 16.0). Compound **4** has four chiral carbon atoms, all with R configurations, indicating that the molecule lacks a plane of symmetry. However, ^1^H and ^13^C NMR analysis of compound **4** shows that, in terms of chemical shifts, it behaves like a symmetrical molecule, with the two branches equidistant from the center showing identical chemical shifts. Therefore, atoms in the chain are numbered and colored the same to indicate similarity, as in compound **4** in Scheme 3. The carbon atoms and their associated hydrogen atoms in the first ethylene unit of the six branches displayed clearly distinct chemical shifts. Yet, the ^1^H and ^13^C signals of the second ethylene units in all six branches were very similar to each other, suggesting that atoms farther from the core are not significantly affected by the electronic environment of the core.

Compound **4** (G0.5), which contains six azido groups, was treated with the surface BB (**3b**) to synthesize G1 (compound **5**) via copper-catalyzed alkyne-azide click chemistry (step b). In copper-catalyzed alkyne-azide click reactions in which copper ions, such as CuI or CuSO_4_ with vitamin C, are used as catalysts, copper contamination is a common issue. Since our dendrimers have numerous electronegative atoms in their branches (linkers), their internal cavities formed by these branches can trap copper ions. This trapping can lead to copper contamination, which can cause issues across various applications. In our previous study, we found that using copper metal granules (99.99%, 100 mesh) significantly reduces copper contamination in the target dendrimers [34]. However, copper metal granules are much less efficient catalysts than copper ions. Therefore, to facilitate the conversion, the reaction was carried out in a microwave reactor set at 150 W and 77 °C. The attachment of six BBs to form **5** was completed within 7 h, as shown by LC/MS analysis. This high efficiency is likely due to the unique arrangement of OH groups in the D-mannitol core, which causes the branches of **4** to stagger and reduces steric hindrance, allowing antioxidant BBs easy access to the branch termini. Although the copper-chelated target compound was not detected in the MS analysis, the reaction mixture was treated with saturated aqueous EDTA to ensure the target compound was free of copper contamination, followed by solvent removal and subsequent work-up with a chloroform-water mixture. Then, the reaction mixture was purified using a size-exclusion column (LH-20), with methanol as the eluent. The purified compound was analyzed by LC/MS. Compound **5** appeared as a multiply charged species, [M + 2H]^2+^ to [M + 6H]^6+^, due to its large size (Supplementary Figure S14). The monoisotopic masses of these charge states were deconvoluted to a mass of 3183.4558 amu, which closely matches the calculated exact mass of 3183.4416 amu. The G1 dendrimer was also characterized using ^1^H and ^13^C NMR (Supplementary Figures S15 and S16), along with 2D correlation NMR. As mentioned above, the atoms beyond the first ethylene units in all six branches exhibit identical chemical shifts in NMR analysis.

**Scheme 4:**
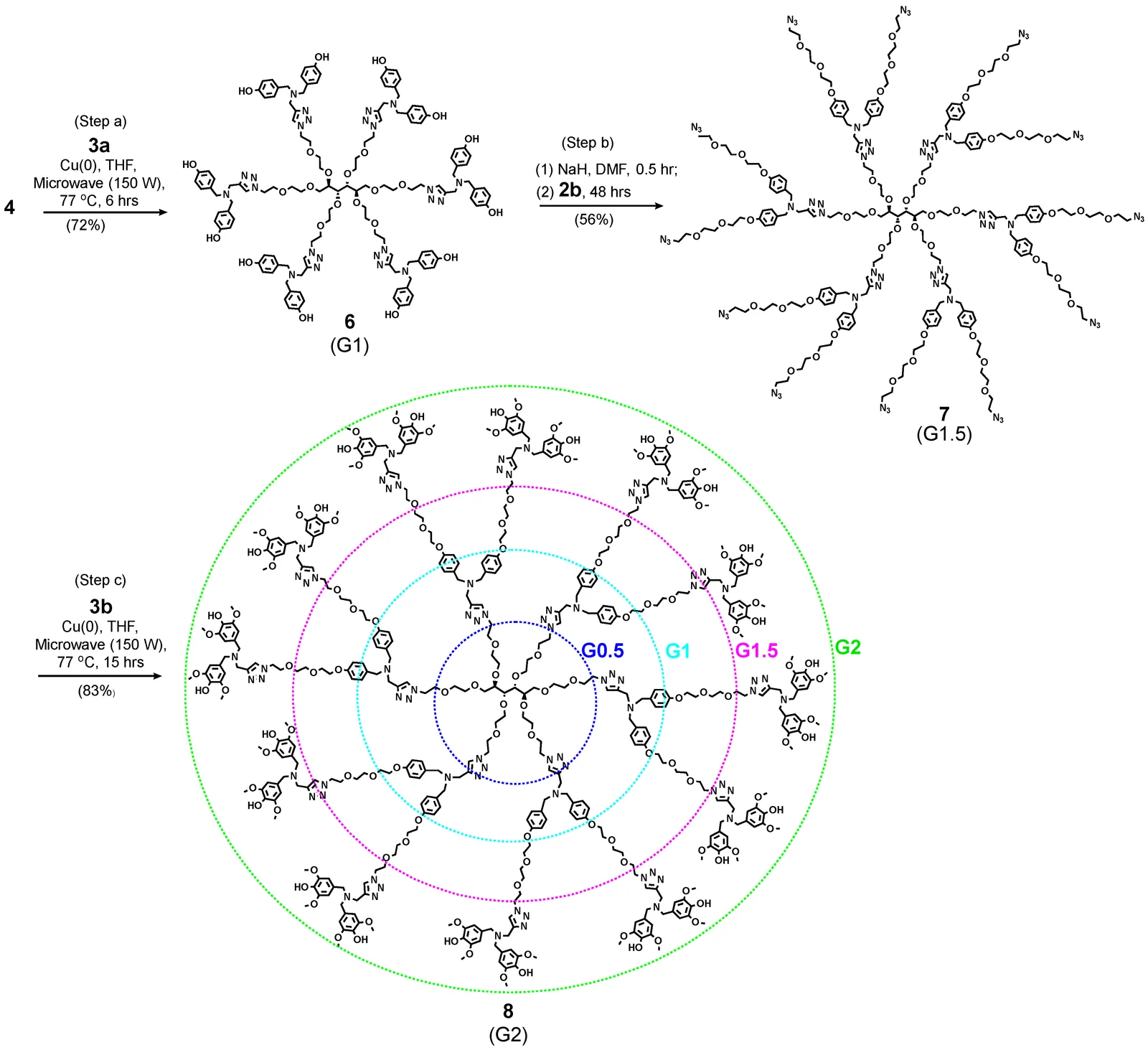
Synthesis of the G2 target dendrimer. The color-coded dotted circle indicates the approximate range of each generation.

To move forward with the synthesis of the G2 dendrimer, another type of G1 (compound **6**) was first synthesized by reacting compound **4** with the internal BB (compound **3a**) using the alkyne-azide click reaction (step a, Scheme 4), the same method that produced compound **5**. The reason for using compound **3a** over **3b** as the internal BB was to minimize steric hindrance when attaching the linker **2b** to the OH termini in the following step b. The purified compound **6** was analyzed with MS, showing multiply charged states ranging from [M + H]^+^ to [M + 6H]^6+^, corresponding to average masses of 2465.1941 amu to 411.7084 amu (Supplementary Figure S17). Their monoisotopic masses were deconvoluted to a mass of 2463.1927 amu, close to the calculated exact mass of 2463.1881 amu. The compound was characterized using ^1^H and ^13^C NMR (Supplementary Figures S18 and S19), along with 2D correlation NMR.

Compound **6** was reacted with **2b** to produce compound **7** (G1.5) (step b). This process was performed in a stepwise manner: First, compound **6** was treated with sodium hydride (NaH) in anhydrous DMF for 0.5 h. After this initial step, linker **2b**, which is a longer linker than **2a**, was added. During our study, we found attaching 12 equivalents of surface BBs to G1.5 carrying 12 shorter linkers was challenging due to steric hindrance between the incoming BBs, leading to lower yields. As a result, the longer linker **2b** was used to minimize steric hindrance during the surface BB attachment to the azido termini of G1.5 and to enhance reaction efficiency. The reaction was completed in two days. With bases like K_2_CO_3_, DBU, and DIPEA, the reaction did not reach completion even after two weeks. The compound was purified on silica gel using hexane-acetone (1:0 to 0:1). Despite its large size, it eluted quickly and was easily purified. Compound **7** was analyzed on MS, which showed [M + 2H]^2+^ to [M + 6H]^6+^, with average masses ranging from 2176.1124 amu to 726.0449 amu (Supplementary Figure S20). Their monoisotopic mass after deconvolution is 4348.2090 amu, while the calculated mass is 4348.2096 amu. Compound **7** was also characterized via NMR. The signal assignments were carried out based on chemical shifts, splitting patterns, and integrations in ^1^H and ^13^C NMR spectra (Supplementary Figures S21 and S22) and in 2D correlation NMR spectra, which were compared with the estimated spectra. Since the core’s influence on the chemical shifts of atoms ended at the fourth atom in the added branches in G0.5, the six branches exhibited the same chemical shifts for the newly added functional groups in all subsequent generations beyond G0.5. The ^1^H and ^13^C signals of G1.5 were assigned by first identifying signals present in its precursor, compound **6**. The new peaks in G1.5 originate from linker **2b**, with each CH_2_ signal integrating to 24H and splitting into triplets or multiplets depending on its environment.

**Figure 1:**
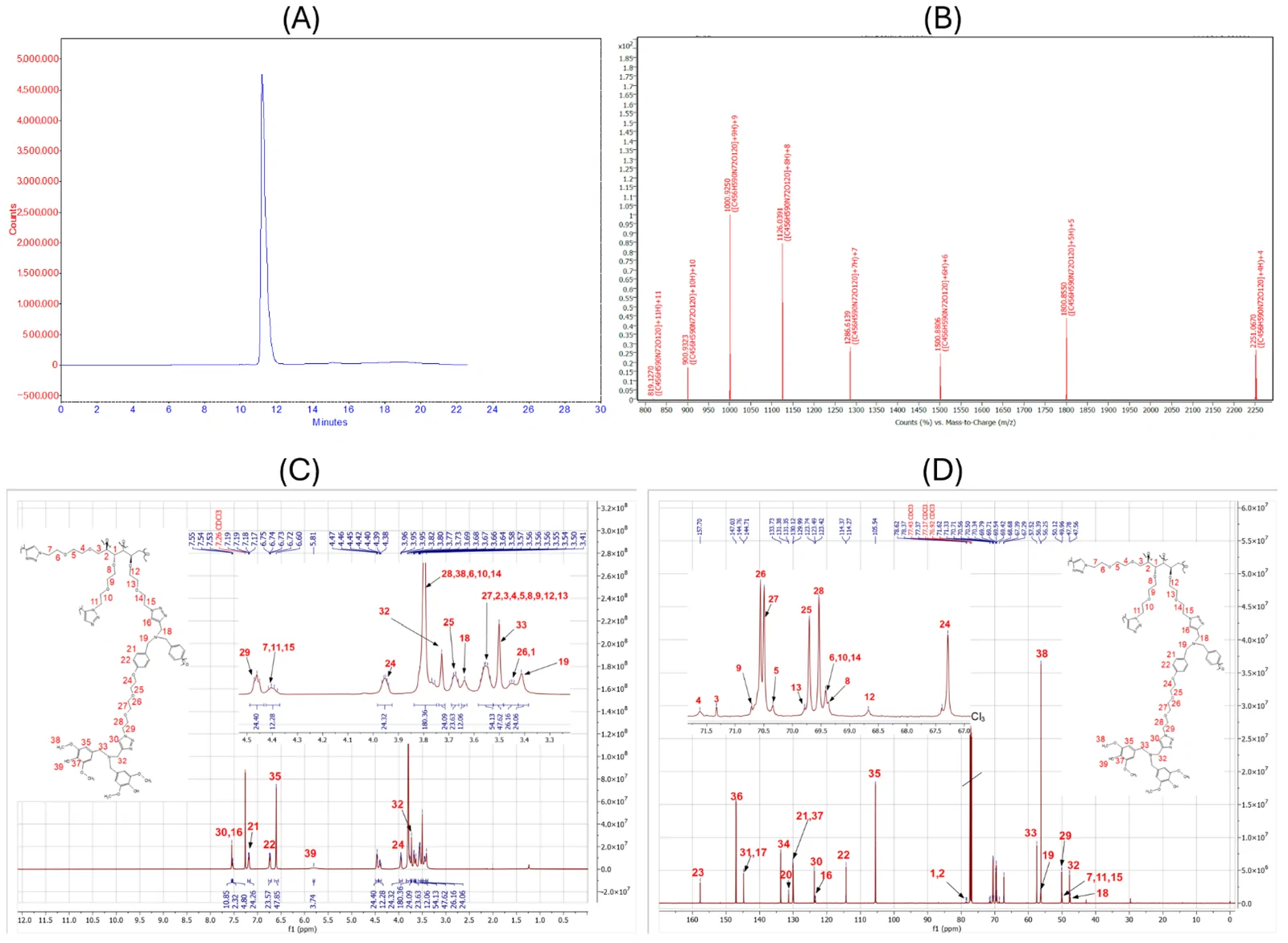
Compound **8** (G2) analysis: (**A**) HPLC chromatogram; (**B**) MS spectrum; (**C**) ^1^H NMR; (**D**) ^13^C NMR.

Generation 2 dendrimer (compound **8**) was synthesized using a microwave-assisted click reaction of compound **7** with twelve equivalents of surface BBs (compound **3b**) in anhydrous THF, with copper granules serving as the catalyst (step c). Reaction was monitored using LC/MS and required 15 h at 150 Watts and 77 °C. Upon completion, the reaction mixture was filtered through Celite to remove copper granules. The filtrate was then treated with a saturated EDTA solution and stirred for 1 h to remove any remaining copper. The solution was then dried and partitioned between chloroform and water. The dried chloroform layer was purified using a size-exclusion column with CHCl_3_/MeOH (90:10). HPLC analysis confirmed that no other impurities were present in the purified product (Figure 1A). In the LC/MS analysis (Figure 1B), the macromolecular G2 dendrimer was observed in charged states from [M + 4H]^4+^ to [M + 11H]^11+^, deconvoluted to 8994.1968 amu, compared to the calculated mass of 8994.2279 amu.

The NMR spectra of G2 were more complex than those of its precursors. As the dendrimer generation increased, the interior ^1^H and ^13^C signals, especially those at the core, became progressively weaker. The hydrogen signals became noticeably broad. To assist in assigning proton and carbon signals (Figure 1C,D), correlation 2D NMR experiments, including ^1^H-^1^H COSY, ^1^H-^13^C HSQC, ^1^H-^13^C HMBC, and ^1^H-^1^H NOESY, were performed (Supplementary Figures S23–S26). Most G1.5 signals were identified in the G2 spectrum, except for the signal of CH_2_ groups next to the terminal azido groups in G1.5. This signal shifted from 3.23 ppm to around 4.5 ppm (Figure 1C) in G2, since the azido group participated in forming a new triazole ring with the alkyne functionality of the BB **3b**. The new signals in G2 include a CH (labeled as 30) within the new triazole ring, which integrates for 12Hs, and a CH_2_ group (labeled as 32) with an integration of 24Hs. The signals for the surface units in the G2 dendrimer resemble those for similar units in the G1 (compound **5**) in terms of chemical shifts and splitting patterns. However, peak integrations in G2 are twice as large as in G1 because the number of surface units in G2 has doubled compared to G1. Our analysis using LC/MS and NMR confirmed the successful synthesis of the target G2 dendrimer, which features twenty-four syringic groups.

### 2.2. DPPH Assay Results

The synthesized dendrimers were evaluated for their radical scavenging antioxidant effects using the DPPH assay (Table 1). The IC_50_ values for the dendrimers were compared to those for the syringaldehyde starting material and BHT, a popular food preservative, which served as comparison controls.

IC50 values for G2 (compound **8**) and G1 (compound **5**) were 0.7 μM and 1.36 μM, respectively. The IC50 for the syringaldehyde and BHT were 260 μM and 880 μM, respectively. G2 with 24 syringic units exhibits DPPH radical scavenging activity 371 times greater than that of syringaldehyde and is 1257 times more potent than BHT. G1 with 12 syringic units is 191 times more effective than syringaldehyde and 647 times more potent than BHT in radical scavenging. G2 is approximately twice as effective as G1. Both dendrimers greatly exceed the expected 24- and 12-fold improvements over their single-unit counterparts. Even though the number of syringic units on dendrimers is accounted for, the G2 dendrimer still remains 15 times more effective than syringaldehyde and 52 times more potent than BHT. Similarly, G1 is 16 times more efficient than syringaldehyde and 54 times more potent than BHT.

### 2.3. β-Carotene Bleaching Assay Results

**Figure 2:**
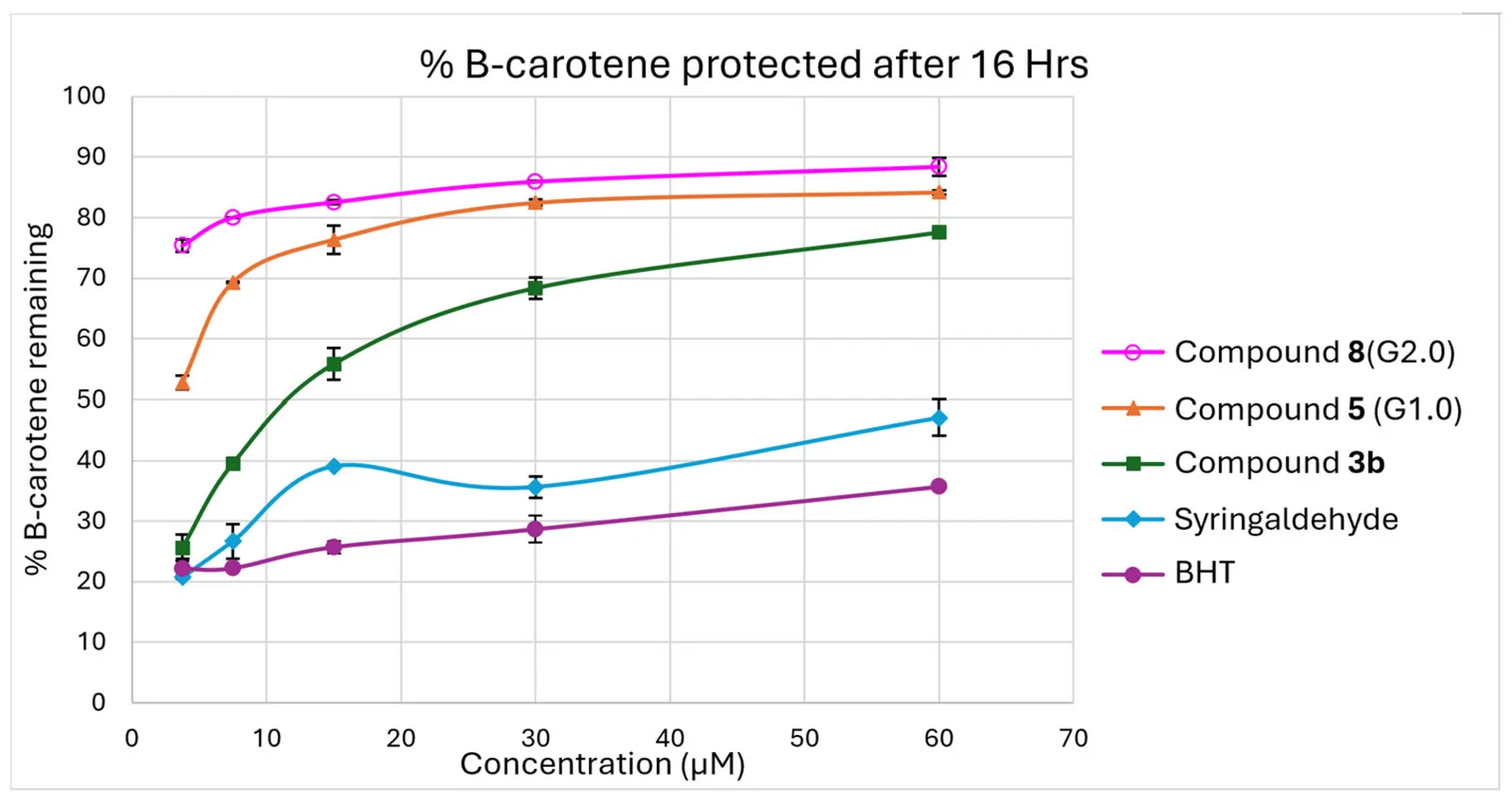
Effective protection of β-carotene from bleaching by dendritic antioxidants. A one-way ANOVA was conducted to compare the effective protection results across the test compounds, revealing a statistically significant difference between compounds and within each compound (*F*(4, 20) = 18.74, *p* = 1.53 × 10^−6^). The error bar is almost invisible when the standard error of the mean results is very small.

To evaluate the relative antioxidant activities at equal concentrations, the β-carotene bleaching assay was employed, following a previously reported method [35]. In this assay, linoleic acid undergoes autoxidation, producing peroxyl radicals that react with β-carotene, leading to its degradation and the fading of its characteristic orange color. Antioxidants help delay this bleaching process by neutralizing radicals. Five different concentrations (3.75 μM, 7.5 μM, 15 μM, 30 μM, and 60 μM) were tested, with samples incubated at 45 °C in the dark for 16 h, and their absorbance measured at 470 nm. Then, the percentage of β-carotene remaining over time (Figure 2) was calculated.

As observed in the DPPH assay, G2 provided significantly better protection of β-carotene compared to G1 across all tested concentrations (3.75 μM to 60 μM). It preserved 75% to 88% of β-carotene after 16 h of incubation and exhibited a relatively flat dose-response curve, indicating it reaches maximum effectiveness at low doses and is a highly potent antioxidant. Under the same conditions, G1 protected 51% to 84% of β-carotene in a dose-dependent manner. In comparison, syringaldehyde and BHT offered significantly lower protection within the same concentration range, ranging from 21% to 47% and 22% to 36%, respectively.

### 2.4. Cell Viability Assay Results

**Figure 3:**
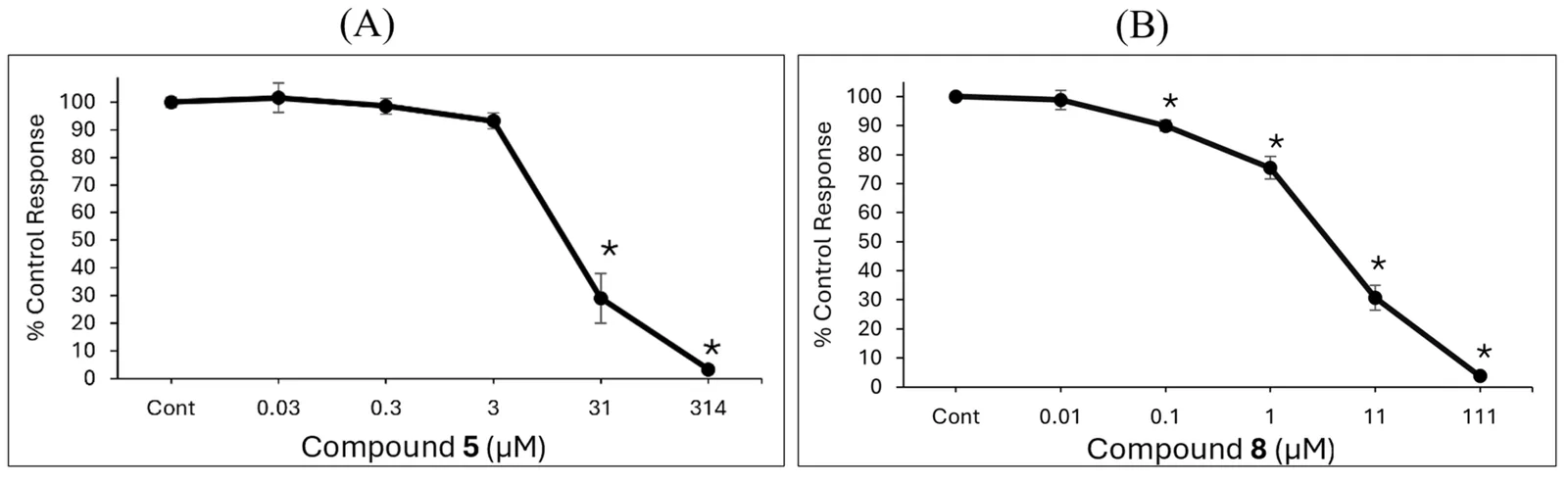
RAW 264.7 macrophage cell viability following 24 h treatment with (**A**) Compound **5** (G1) and (**B**) Compound **8** (G2). The asterisk (*) indicates a significant difference from the untreated control (*p* < 0.05).

Cell viability of the dendrimers was evaluated using RAW 264.7 macrophage cells, which are commonly used as an in vitro model to study inflammatory responses. Macrophage cells are the first cells to respond to infection by releasing reactive oxygen and nitric oxide (NO). Chemokine release by macrophages leads to activation of additional immune cells and a more comprehensive response. Our initial studies have assessed the effect of dendritic compounds on macrophage cell viability. Cells were exposed to compound **5** (0–314 µM) or compound **8** (0–111 µM) for 24 h. Compound **5** (G1) did not affect cell viability at a concentration of 3 µM and below, but decreased viability significantly at concentrations of 31 µM and 314 µM by 71% and 96.8% respectively (Figure 3A). In comparison, compound **8** (G2) showed a gradual decrease in cell viability at a test concentration as low as 0.1 µM (Figure 3B). The decreased levels at concentrations of 0.1 µM, 1 µM, 11 µM, and 111 µM were 11.1%, 24.6%, 69.3%, and 96.3%, respectively. The LD_50_ for compound **5** was 15 µM, and for compound **8**, it was 4 µM. The reason for this difference in toxicity is unclear but may reflect the size difference between these compounds. Size may influence cellular interactions at the membrane and ultimately uptake into the cell.

## 3. Discussion

Based on the DPPH and β-carotene-bleaching assays, G2 with 24 phenol rings is a stronger antioxidant than G1 with 12 phenolic units, suggesting that increasing the number of antioxidant units on the dendrimer surface improves their effectiveness as antioxidants. In other words, larger antioxidants are more efficient than smaller counterparts, provided that the number of their antioxidant phenolic units increases as their size grows. Although it remains uncertain whether our findings with G1 and G2 antioxidant dendrimers can be extrapolated to other radical scavenging systems until further research is conducted, our current study results are consistent with other previous size-related antioxidant activity studies [36,37].

For macromolecular antioxidants carrying multiple phenolic units to be highly potent, they should meet the correct structural requirements for antioxidants. Given the fact that syringaldehyde produces weak antioxidant properties by itself, high antioxidant activity would not be expected from simply combining 12 syringaldehyde units or 24 units. Highly effective antioxidant activities against DPPH and peroxyl radicals from these antioxidants are produced by strategically attaching multiple units of syringaldehyde to a dendrimer scaffold designed with an optimal number of branching units. The main difference between before and after attaching syringaldehyde to the dendrimer surface is the presence of an aldehyde group versus a benzylic group. Aldehyde is electron-withdrawing by nature, whereas the benzylic group is weakly electron-donating. As reported in previous studies, electron-donating groups promote antioxidant activity by stabilizing radicals formed on the antioxidant after it donates its electron(s) to the radical(s) [30,31,32,33]. This indicates that converting the functional group from electron-withdrawing to electron-donating enhances the antioxidant potential of syringaldehyde. In other words, the use of reductive amination, in which an aldehyde is transformed into a benzylic group, allows syringaldehyde to function as a phenolic unit with essential features of a strong antioxidant, including electron-donating groups (EDGs) at both the *ortho* and *para* positions. It is our assertion that these distinctive structural features of the syringic units are instrumental in contributing to the high antioxidant activity observed in these dendrimers. Additionally, having multiple syringic units on the surfaces of dendrimers can further enhance their antioxidant potential, possibly through so-called dendritic cooperative effects. These effects arise from the unique branched structure and multivalent surface functionality of dendrimers [38]. The close spatial arrangement of multiple syringic units on the dendrimer surface may facilitate electronic communication between adjacent syringic moieties, thereby enabling cooperative electronic effects. This interaction can lower the O–H bond dissociation enthalpy, making it easier for these dendrimers to donate hydrogen atoms and neutralize free radicals. However, it is important to note that dendritic effects are not always positive. Negative dendritic effects can also occur if the phenolic units contain improper functional groups—such as electron-withdrawing groups positioned *ortho* to their hydroxyl (OH) groups [31]. Additionally, we observed cases in which dendrimers with multiple phenol rings showed no dendritic cooperative effects at all. For example, phenols lacking any electron-donating group *ortho* to the phenolic OH did not exhibit detectable antioxidant activities [29]. For this reason, a G2 dendrimer with compound **3a** (internal BB) was not synthesized in this study.

Our current results suggest that larger antioxidants, which contain more phenolic units, are more effective than smaller antioxidants with fewer phenolic units. However, it’s important to note that many previous studies have shown otherwise. These studies involve antioxidants of varying molecular weights, making it difficult to establish a clear threshold that distinguishes small from large antioxidants. Some research indicates that small antioxidants (based on molecular weight, not particle size) perform better than large antioxidants [39,40,41]. Conversely, other reports suggest that medium-molecular-weight antioxidants can outperform both small and large ones [42]. This implies that molecular size does influence antioxidant effectiveness. Ultimately, the optimal size may depend on the specific delivery method, application, and testing parameters [41,43]. Overall, small antioxidants exhibit high antioxidant activity because they encounter less steric hindrance, allowing them better access to target radicals [44,45,46]. In cellular environments, small antioxidants may show higher activity than larger ones because of better membrane permeability, solubility, and diffusibility, leading to improved cellular uptake and bioavailability [45,47,48]. However, small antioxidants are rapidly consumed after scavenging a free radical [49], can readily undergo degradation [50], and tend to be less potent than larger antioxidants [28,29].

Large-molecular-weight antioxidants, such as dendrimers, polymeric antioxidants, PEGylated phenols, and protein-based systems, are often preferred in certain applications, including biomedical, cosmetic, and food industries, where enhanced stability, prolonged action, and controlled release are important [51,52,53,54,55]. The notable benefits of large antioxidants over their low-molecular-weight counterparts include enhanced thermal and oxidative stability [56], resulting in greater resistance to degradation caused by heat, light, and oxygen due to steric hindrance and shielding effects, which lead to notably improved antioxidant properties and greater shelf stability [57]. More importantly, these large antioxidants can be used to encapsulate other molecules, such as drugs, within their internal cavities or to attach targeting moieties to their surfaces, enabling selective delivery of their cargos to the target site and resulting in both therapeutic and antioxidant effects [18,58,59,60,61,62]. Based on these size-related antioxidant studies, the optimal size of antioxidants varies by application, indicating that antioxidants of various sizes are all beneficial.

According to our cytotoxicity data, the antioxidant’s size matters, and the larger dendrimer appears more toxic than the smaller one. However, previous studies reporting the relationship between the size of nano-antioxidants and their cytotoxicity generally show an inverse correlation: smaller nanoparticles tend to exhibit higher cytotoxicity [63,64]. Studies consistently show that as nanoparticle size decreases, their surface area-to-volume ratio increases, leading to enhanced cellular uptake and higher generation of reactive oxygen species, which results in increased oxidative stress and cytotoxic effects. Nanoparticles below certain sizes can more readily infiltrate cells and interact with intracellular components, amplifying apoptotic or autophagic pathways associated with oxidative stress [65]. These indicate that smaller nano-antioxidants induce greater cell damage [66,67]. Since the molecular composition of the nanoparticles used in earlier studies differs from that of our dendrimers, the variation in size-related cytotoxicity between their findings and ours is not entirely surprising. Future studies comparing subcellular localization may provide additional insights into this discrepancy. Nevertheless, minimizing cytotoxicity should be a primary consideration in the design of nano-antioxidants, as excessive toxicity can limit their viability for various applications. It is crucial to optimize size to reduce cytotoxicity while maintaining antioxidant activity. Based on the literature, size is just one factor that influences cytotoxicity; particle type, shape, and surface properties also play significant roles [64]. Additionally, the same particle can exhibit different toxic effects depending on the specific cell type involved [63]. Another contributing factor to cytotoxicity, which may be relevant to our dendrimers, is hydrophobicity [68,69,70]. The increased cytotoxicity of G2 may be attributed to its higher number of hydrophobic phenolic units on the surface compared to G1. According to literature, an effective way to improve the toxicity profile of our dendrimers may involve modifying their surfaces with functional groups known to lower cytotoxicity, such as glycerol, polyethylene glycol, or dextran [21,71].

Cytotoxicity may differ depending on cell types. Therefore, we are currently testing these compounds in different cell lines to evaluate their overall cytotoxicity and anti-inflammatory effects across various cell types, with the aim of exploring their potential applications. Future studies will involve how to strategically modify the surface of these dendrimers to reduce their toxicity profile without compromising their antioxidant effects.

## 4. Materials and Methods

### 4.1. Chemicals

D-mannitol, BHT, sodium triacetoxyborohydride (NaBH(OAc)_3_), 2-picoline borane, sodium azide, p-toluenesulfonyl chloride, sodium hydride in powder, 1,8-diazabicyclo [5.4.0]undec-7-ene (DBU), *N*,*N*-diisopropylethylamine (DIPEA), triethylamine (TEA), 2,2-diphenyl-1-picrylhydrazyl (DPPH), and ethylenediaminetetraacetic acid (EDTA) disodium salt dihydrate were purchased from Sigma Aldrich (Milwaukee, WI). Syringaldehyde, 2-(2-chloroethoxy)ethanol, and 2-[2-(2-chloroethoxy)ethoxy]ethanol were obtained from AmBeed Inc. (Buffalo Grove, IL, USA). All chemicals were used as received without further purification. All reaction solvents, as well as deuterated NMR solvents, were purchased from VWR (Visalia, CA, USA). LC-MS grade acetonitrile was sourced from Honeywell (Lodi, NJ, USA). LC-MS grade formic acid was purchased from Thermo-Scientific (Waltham, MA, USA). Ultrapure water was obtained from our Milli-Q UVPlus unit (Millipore, France). LH-20 was purchased from HongKong iPure Biology CO. Ltd. (HongKong, China). Cell lines were purchased from ATCC (Manassas, VA, USA). Cell culture reagents were purchased from Sigma Aldrich (St. Louis, MO, USA).

### 4.2. General Information for Synthesis and Purification

Copper-catalyzed alkyne-azide click reactions were carried out in a CEM (Discover 2.0) microwave reactor (Matthews, NC, USA). Copper metal granules (99.99%, 100 mesh) were soaked in a 20% sodium hydroxide solution for 30 min, followed by rinsing with water. They were subsequently soaked in a 20% sulfuric acid solution for 30 min, thoroughly rinsed with water, rinsed with acetone, and finally dried. The dried copper granules were stored under argon. Reaction conditions are detailed in the synthesis section of each compound.

For purification, we used either silica gel column chromatography with a commercially available pre-packed silica gel column (40 g, 230–400 mesh, Luknova, Mansfield, MA, USA) and the CombiFlash Companion (Teledyne Isco) system, or size exclusion chromatography with Sephadex LH-20 (50 g).

Fractions containing the target compounds were analyzed with a standalone HPLC system (Hitachi, Japan) to assess the purity of each fraction and combine similar ones. The mobile phase was a gradient of acetonitrile and water, ranging from 5% to 95% acetonitrile with 0.1% trifluoroacetic acid. The flow rate was 1 mL/min, and analytes were detected at 214 nm.

### 4.3. Characterization

^1^H NMR spectra were recorded on a 500 MHz Bruker NMR (Billerica, MA, USA). Chemical shifts (δ) are reported in ppm. The NMR solvent for each compound was indicated in the synthesis section. The concentration of the NMR samples was 20 mg/mL. Coupling constants (J) are given in Hz. The multiplicities of signals are denoted as follows: s = singlet, br.s = broad singlet, t = triplet, dd = doublet of doublet, dtd = doublet of triplet of doublet, t+t = triplet overlapped with another triplet, m = multiplet. ^13^C NMR spectra were recorded on a 125 MHz Bruker NMR (Billerica, MA, USA). ^1^H NMR and ^13^C NMR spectra for each compound are shown in Supplementary Materials.

Mass spectra were obtained using either an ultra-performance liquid chromatography (UPLC)–electrospray ionization (ESI)–quadrupole time-of-flight (Q-TOF) mass spectrometer (Agilent, AdvanceBio 6545XT) (Santa Clara, CA, USA) or a high-performance liquid chromatography (HPLC)–electrospray ionization (ESI)–time-of-flight (TOF) mass spectrometer (Agilent, G6230B) (Santa Clara, CA, USA). Both the UPLC and HPLC systems are equipped with a multi-wavelength diode array detector.

The LC/MS samples were prepared at a concentration of 1 ng/mL. For fractions obtained through column chromatography, samples were created by performing three serial dilutions using the matrix (water–acetonitrile = 50:50, with 0.1% formic acid). Before injection, all samples were filtered through a syringe filter (Minisart RC4 cellulose membrane, 0.2 μm pore size) purchased from Sartorius (Epsom, Surrey, UK). The injection volume was 0.3 µL. LC separations were conducted on an Agilent C18 column (InfinityLab Poroshell 120 EC-C18, with a mean particle size of 1.9 μm, 2.1 mm inner diameter, and 50 mm length) utilizing a water–acetonitrile gradient system (from 5% to 95% acetonitrile) containing 0.1% formic acid. The flow rate was 0.4 mL/min for 10 min, with the column oven kept at 35 °C. Mass analysis of all samples was performed using the Dual Agilent Jet Stream Electrospray Ionization (AJS ESI) as the ion source under these conditions: gas temperature at 320 °C, drying gas flow rate at 8 L/min, nebulizer gas at 35 psi, sheath gas temperature at 350 °C, sheath gas flow at 11 L/min, capillary voltage at 3500 V, nozzle voltage at 1000 V, fragmentor voltage at 120 V, skimmer voltage at 65 V, MS range from 100 to 3200 *m*/*z*, and an acquisition rate of 1 spectrum per second. Tuning and calibration of the instrument were carried out using an ESI-L low-concentration tuning mix and hexamethoxyphosphazine (0.1 mM HP-0321) purchased from Agilent.

### 4.4. DPPH Assay

Antioxidant activity was assessed using standardized methods with minor modifications [72]. DPPH solution was prepared in methanol at 0.0916 mM. All antioxidants were dissolved in 10% PEG600 in methanol. We prepared antioxidant concentrations at 0.2–0.003125 mM for compound **8** (G2.0), 0.3–0.002344 mM for compound **5** (G1), 50–0.78125 mM for syringaldehyde, and 300–4.6875 mM for BHT. A volume of 25 μL of antioxidant solution or 10% PEG in methanol (used as blank) was added to 1.2 mL of DPPH reagent. The final antioxidant concentrations ranged from 4.17 to 0.065 μM for compound **8** (G2), 6.25 to 0.049 μM for compound **5** (G1), 1041.67 to 16.27 μM for syringaldehyde, and 6250 to 97.65625 μM for BHT. Samples were incubated at room temperature in the dark for 1 h before measuring absorbance at 515 nm. All experiments were performed in triplicate, with the coefficient of variation for % inhibition remaining below 6%. The IC50 value of each antioxidant was determined from its graph, which plotted concentration against % DPPH remaining, calculated as 100 − (((Absorbance of Blank − Absorbance of Sample)/Absorbance of Blank) * 100).

### 4.5. β-Carotene Bleaching Assay

The ability of the test compounds to protect β-carotene from bleaching was determined using the established method with some modifications [35]. The reagent was prepared by dissolving 4 mg of β-carotene in 100 mL of chloroform. Then, 0.5 mL of linoleic acid was added and thoroughly mixed by means of vortexing, resulting in an orange-colored homogeneous solution. Stock solutions of 500 µM for all test compounds were prepared individually and serially diluted to create concentrations ranging from 250 to 31.25 µM. A 120 µL sample solution was added to 1000 µL of the reagent, vortexed, and the mixture was incubated at 45 °C in a brown glass vial with a cap throughout the test period. Absorbances were measured at time zero (corresponding to the blank with 1000 µL reagent and 120 µL chloroform) and at 1, 3, and 16 h at 470 nm for all test compounds. The test was performed three times with duplicates for each compound. The percentage of remaining β-carotene was plotted against concentration.

### 4.6. Cell Viability Assay

RAW 264.7 murine macrophage cells were cultured in RPMI1640 media supplemented with 10% fetal bovine serum and 2 mM glutamine at 37 °C, in a 5% CO_2_ humidified incubator. To assess viability, 100 µL of cells (5 × 10^5^/mL) were added to 96-well plates and incubated with 100 µL media control or test compound at varying concentrations for 24 h. A volume of 20 μL of a 5 mg/mL 3-(4,5-dimethylthiazole-2-yl)-2,5-diphenyltetrazolium bromide (MTT) solution in 0.01 M phosphate-buffered saline was added to each well 2 h before termination of the experiment. The plates were centrifuged (450× *g*, 10 min) and supernatants were removed. The resultant formazan crystals were dissolved in 100 µL DMSO and absorbance was measured using a Biolog microplate reader (Biotek Instruments) at dual wavelengths 590 nm and 650 nm. Percent control response was calculated (absorbance of treatment/absorbance of control × 100). All experiments were performed in triplicate and repeated.

### 4.7. Synthesis Methods and Analysis Results

#### 4.7.1. Compound **2a**

Compound **2a** was made according to previously published methods [29].

#### 4.7.2. Compound **2b**

2-[2-(2-Chloroethoxy)ethoxy]ethanol (10 mL, d = 1.16 g/mL, MW = 168.62 g/mol, 68.79 mmol) and sodium azide (4.9195 g, 65.01 g/mol, 75.67 mmol) were dissolved in DMF (200 mL). Water (2 mL) was added to the mixture. The reaction mixture was stirred for 48 h at 60 °C. Then, the reaction mixture was filtered via vacuum filtration, and the DMF filtrate was dried using a rotary evaporator with a vacuum pump. The resulting whitish liquid was mixed with acetone to precipitate all the sodium chloride byproduct and leftover sodium azide, followed by filtration and rotary evaporation. This process was repeated until no more solid residues remained in the dried material. The dried material (compound **1b**, 13.96 g, 175.10 g/mol, 79.73 mmol) in the round-bottom flask was transferred to a 500 mL three-neck round-bottom flask cooled in a dry-ice bath containing dry ice in isopropanol. Chloroform (20 mL) was used to rinse out the remaining material. Then, triethylamine (18 mL, 0.726 g/mL, 101.06 g/mol, 129.31 mmol) was added to the stirring solution. In a beaker, p-toluenesulfonyl chloride (16.6672 g, 190.84 g/mol, 0.08734 mol) was dissolved in chloroform (250 mL). Then, the solution was transferred to an addition funnel and added to the reaction dropwise. The reaction mixture was kept in the dry ice bath for 1 h and then allowed to warm up to room temperature. It was stirred for 24 h, after which it was transferred to a round-bottom flask and dried on a rotary evaporator to approximately half its original volume. Then, the solution was washed with water three times. The chloroform layer was dried with magnesium sulfate, followed by filtration and evaporation. The dried neat material was directly loaded onto a pre-packed silica gel column (40 g) and purified using a hexane-ethyl acetate solvent system (9:1).

Yield: 88% (19.92 g); Appearance: clear oily liquid; R*_f_* = 0.43 in hexane-ethyl acetate (1:1); ^1^H NMR (500 MHz, CDCl_3_) δ 2.07 (s, ^1^H), 2.35 (s, ^3^H), 3.27 (t, *J* = 5.1 Hz, ^2^H), 3.41–3.52 (m, ^4^H), 3.55 (t, *J* = 5.0 Hz, ^2^H), 3.60 (q, *J* = 5.2 Hz, ^2^H), 4.02–4.13 (m, ^2^H), 7.28 (s, ^2^H), 7.70 (d, *J* = 8.3 Hz, ^2^H); ^13^C NMR (126 MHz, CDCl_3_) δ 21.68, 50.71, 68.81, 69.33, 70.12, 70.65, 70.83, 128.01, 129.89, 133.04, 144.90; LC/ESI-TOF MS: *m*/*z* calculated for [M + Na]^+^, C_13_H_19_N_3_O_5_SNa, is 352.0943 amu; found as 352.0986 amu.

#### 4.7.3. Compound **3a** (Internal BB)

4-Hydroxybenzaldehyde (2.30 g, 122.12 g/mol, 18.83 mmol) was dissolved in methanol, and then propargylamine (1.2 mL, 0.867 g/mL, 55.08 g/mol, 18.89 mmol) was added. The mixture was stirred for 30 min. The reducing agent, 2-picoline borane (1.80 g, 106.96 g/mol, 16.83 mmol), was added to the reaction mixture, which was stirred overnight. Second equivalents of 4-hydroxybenzaldehyde and 2-picoline borane were added to the reaction mixture at a 30-min interval. The reaction continued overnight and was stopped the following day after confirming the formation of the target compound. Methanol was removed from the mixture using rotary evaporation, and the residue was worked up into ethyl acetate and water. Magnesium sulfate was added to the organic layer to trap residual water, then filtered off. The filtrate was rotary evaporated, and the product mixture was kept under house vacuum until ready for purification. The mixture was dissolved in acetone, and the slurry was prepared with silica gel. The dried mixture was loaded onto a silica gel column and purified using a gradient hexane-ethyl acetate system (7:1 → 2:1). The pure fractions were combined based on TLC and LC/MS. The product was left under house vacuum overnight for complete drying, purged with argon gas, and stored in the freezer until ready to use.

Yield = 82% (4.12 g); Appearance = white solid; R*_f_* = 0.39 in hexane-ethyl acetate (1:1);^1^H NMR (500 MHz, Acetone) δ 2.54 (t, *J* = 2.4 Hz, ^1^H), 3.05 (d, *J* = 2.5 Hz, ^2^H), 3.42 (s, ^4^H), 6.65–6.68 (m, ^4^H), 7.05–7.08 (m, ^4^H), 8.08 (s, ^2^H); ^13^C NMR (126 MHz, Acetone) δ 40.44, 56.63, 74.22, 78.60, 115.24, 129.86, 130.30, 156.63; LC/ESI-TOF MS: *m*/*z* calculated for [M + H]^+^, C_17_H_18_NO_2_, is 268.1332 amu; found as 268.1337 amu.

#### 4.7.4. Compound **3b** (Surface BB)

Syringaldehyde (3.55 g, 182.17 g/mol, 19.49 mmol) was dissolved in methanol. Propargylamine (1 mL, 0.867 g/mL, 55.08 g/mol, 15.74 mmol) was then added to the dissolved syringaldehyde. The mixture was stirred for 30 min, after which 2-picoline borane (1.80 g, 106.96 g/mol, 16.83 mmol) was added. The reaction was allowed to run overnight with continuous stirring. The next day, a second equivalent of syringaldehyde and 2-picoline borane was added at a 30-min interval. The reaction continued overnight. After confirming the formation of the target compound with LC/MS, the mixture was rotary evaporated, dissolved in chloroform, and washed with water. Anhydrous magnesium sulfate was added to the chloroform layer to remove residual water. After filtration, the filtrate was rotary evaporated again and kept under house vacuum until ready for purification. A prepacked 40 g silica gel column was treated with 100 mL of hexane-triethylamine (200:1), followed by 100 mL of hexane. The reaction mixture was dissolved in 5 mL of chloroform, loaded onto the column, and purified using a gradient of hexane-ethyl acetate (5:1 to 3:1). Fractions containing the target compound were combined. The product was eluted along with impurities. The impure target compound was crystallized by dissolving it in a minimal amount of acetone, then diluting the solution with hexane until it remained clear. The clear solution was left at room temperature to allow the target compound to crystallize. The crystals were separated from the colored solution by pipetting out the supernatant. This process was repeated until pure crystals were obtained. LC/MS and TLC were used to identify and assess the purity of the target compound.

Yield = 86% (5.24 g); Appearance = colorless crystal solid; R*_f_* = 0.12 in hexane-ethyl acetate (1:1); ^1^H NMR (500 MHz, Acetone-*d*6) δ 2.61 (t, *J* = 2.4 Hz, ^1^H), 3.16 (d, *J* = 2.4 Hz, ^2^H), 3.45 (s, ^4^H), 3.68 (s, ^12^H), 6.57 (s, ^4^H), 6.91 (s, ^2^H); ^13^C NMR (126 MHz, Acetone-*d*_6_) δ 41.81, 56.60, 57.90, 74.89, 79.52, 107.02, 130.11, 135.96, 148.63; LC/ESI-TOF MS: *m*/*z* calculated for [M + H]^+^, C_21_H_26_NO_6_, is 388.1755 amu; found as *m*/*z* 388.1760 amu.

#### 4.7.5. Compound **4** (G0.5)

D-mannitol (1.00 g, 182.17 g/mol, 5.49 mmol) was weighed into a 250 mL round-bottom flask that had been purged with argon. Then, 50 mL of DMF was added. The mixture was heated to 50 °C to dissolve D-mannitol, with constant stirring for 15 min. The solution was then allowed to cool to room temperature. One scoop of sodium hydride was added, and the mixture was stirred for 30 min to 1 h. A neat compound **2a** (15.00 g, 285.32 g/mol, 52.57 mmol) was added using a cannula needle. The reaction took place over 5–6 days. The reaction mixture was filtered through Celite 545 filter aid, and DMF was removed via rotary evaporation under reduced pressure at 50 °C. Then, the mixture was worked up with ethyl acetate and water. Anhydrous magnesium sulfate was added to the ethyl acetate layer and then filtered out. The ethyl acetate layer containing the target compound was evaporated using a rotary evaporator. The resulting residue was dissolved in 5 mL of chloroform and loaded onto a pre-packed 40 g silica gel column, which was treated with a hexane-TEA (200:1) mixture. Purification was carried out using a hexane-acetone mixture (1:0 to 1:1) containing 1% TEA. The target compound was identified via LC/MS. Fractions were combined based on HPLC analysis, and the product was analyzed using NMR.

Yield = 53% (2.50 g); Appearance = yellow oily material; R*_f_* = 0.53 in hexane-acetone (1:1); ^1^H NMR (500 MHz, Acetone-*d*_6_) δ 3.41 (q, *J* = 4.8 Hz, ^12^H), 3.60–3.68 (m, ^21^H), 3.69 (dd, *J* = 6.8, 3.7 Hz, ^14^H), 3.74–3.91 (m, ^12^H); ^13^C NMR (126 MHz, Acetone-*d*6) δ 51.44, 51.52, 69.53, 70.56, 70.60, 70.61, 70.67, 71.15, 71.39, 71.41, 71.56, 72.54, 79.23, 79.53, 79.57; LC/ESI-TOF MS: exact *m*/*z* calculated for [M + H]^+^, C_30_H_57_N_18_O_12_, is 861.4398 *m*/*z*, found as 861.4431, *m*/*z* calculated for [M + NH_4_]^+^, C_30_H_60_N_19_O_12_, is 878.4663 amu, found as 878.4692 amu, and *m*/*z* calculated for [M + K]^+^, C_30_H_56_N_18_O_12_K, is 899.3957 amu, found as 899.3969 amu.

#### 4.7.6. Compound **5** (G1)

Compound **4** (0.63 g, 860.44 g/mol, 0.73 mmol), compound **3b** (2.52 g, 387.43 g/mol, 6.50 mmol), and copper metal granules (~250 mg) were placed in a 35 mL microwave reaction vessel equipped with a stir bar. Then, anhydrous THF (15 mL) was added via a cannula needle. The vessel was purged with argon gas before use. The reaction was run at 77 °C and 150 W of microwave energy with stirring under N_2_ for 7 h. Upon completion, the mixture was filtered through a Celite 545 filter aid to remove leftover copper granules and blackish fine particles produced during the reaction. The filtrate was treated with a saturated EDTA solution under vigorous stirring for 1 h, and the solution was then dried on a rotary evaporator. The residue was redissolved in chloroform and washed with water 3 times. The combined chloroform layer was dried with magnesium sulfate. After filtering, the filtrate was dried on a rotary evaporator. The resulting residue was then purified using Sephadex LH20 with methanol as the mobile phase. The pure target compound was eluted first. The pure fractions were combined, rotary evaporated, and kept under vacuum overnight before NMR analysis.

Yield = 83% (1.93 g); Appearance = fluffy pale yellow solid; ^1^H NMR (500 MHz, Acetone-*d*_6_) δ 3.42 (dtd, *J* = 23.6, 10.2, 5.6 Hz, ^22^H), 3.51 (s, ^24^H), 3.55 (dd, *J* = 5.7, 3.4 Hz, ^4^H), 3.58–3.65 (m, ^8^H), 3.69–3.73 (m, ^12^H), 3.79 (d, *J* = 1.7 Hz, ^84^H), 4.49 (dt, *J* = 11.0, 5.3 Hz, ^12^H), 6.71–6.75 (m, ^23^H), 7.08 (s, ^5^H), 7.83–7.88 (m, ^6^H); ^13^C NMR (126 MHz, Acetone-*d*6) δ 48.49, 48.54, 50.74, 50.77, 56.78, 58.09, 64.68, 69.30, 70.16, 70.27, 71.10, 71.26, 71.38, 71.53, 72.45, 79.03, 79.45, 107.07, 124.67, 124.70, 130.77, 135.80, 145.33, 145.41, 148.73; LC/ESI-TOF MS: exact *m*/*z* calculated for [M]^+^, C_156_H_206_N_24_O_48_, is 3183.4416 amu; all observed charge states [M + 2H]^3+^ ~ [M + 6H]^6+^ were deconvoluted to 3183.4558 amu.

#### 4.7.7. Compound **6** (G1)

Compound **4** (1.00 g, 860.44 g/mol, 1.16 mmol) was weighed into a 35 mL microwave reaction vessel. Compound **3a** (2.22 g, 267.32 g/mol, 8.31 mmol) was added. Copper granules (300 mg) and a stir bar were added. Anhydrous THF, 15 to 20 mL, was added via a cannula needle and allowed to stir until complete dissolution occurred. The microwave reactor was set to run at 77 °C and 150 W for 6 h under N_2_ gas. LC/ESI-TOF MS was utilized to monitor the reaction and confirm its completion. The mixture was filtered using a Celite 545 filter aid to remove the copper granules. The filtrate was rotary evaporated. The residue was redissolved in 2 mL of acetone and loaded onto a commercially available 40 g pre-packed silica gel column, which was pre-treated with hexane-TEA (200:1), followed by pure hexane (200 mL). Purification was performed using the CombiFlash Companion chromatography system with a hexane-ethyl acetate-methanol gradient solvent system. LC/MS, TLC, and HPLC were employed to identify and confirm the pure fractions, which were then combined, rotated, evaporated, and left under vacuum to dry overnight. The pure compound was analyzed using NMR.

Yield = 72% (2.06 g); Appearance = fluffy pale-yellow material; ^1^H NMR (500 MHz, CD_3_OD) δ ^1^H NMR (500 MHz, MeOD) δ 3.25–3.29 (m, ^2^H), 3.33–3.43 (m, ^49^H), 3.46–3.59 (m, ^12^H), 3.59–3.64 (m, ^12^H), 3.65–3.76 (m, ^13^H), 4.41 (dq, *J* = 10.7, 5.2 Hz, ^12^H), 6.72 (dt, *J* = 8.4, 2.3 Hz, ^24^H), 7.11–7.17 (m, ^24^H), 7.74 (d, *J* = 6.4 Hz, ^6^H); ^13^C NMR (126 MHz, CD_3_OD) δ 47.89, 48.06, 48.46, 49.94, 56.57, 68.20, 68.62, 68.93, 70.00, 70.12, 70.27, 70.35, 71.53, 77.68, 78.12, 114.74, 124.13, 124.21, 129.56, 129.94, 129.97, 144.84, 144.89, 156.12; LC/MS: exact mass calculated for [M]^+^, C_132_H_158_N_24_O_24_, is 2463.1881 amu. Monoisotopic masses for all charged states [M + H]^+^ ~ [M + 6H]^6+^ were deconvoluted to 2463.1927 amu.

#### 4.7.8. Compound **7** (G1.5)

Compound **6** (1.24 g, 2464.20 g/mol, 0.50 mmol) was added to a 250 mL round-bottom flask, followed by the addition of anhydrous DMF via a cannula needle. Once the mixture was completely dissolved, one scoop of sodium hydride was added quickly, and the mixture was stirred for 30 min. Compound **2b** (2.5 g, 329.37 g/mol, 7.59 mmol) was added. The reaction was run for 48 h. Then it was filtered, rotary-evaporated, and worked up into chloroform and water. The target compound was found in the chloroform layer, which was dried with anhydrous MgSO_4_, filtered, and rotary evaporated. The mixture was purified with a gradient system of hexane-acetone (1:0 → 0:1) using the CombiFlash Companion chromatography system. The pure fractions were combined based on the LC/MS and HPLC analysis. Upon complete drying, the pure product was analyzed with NMR.

Yield = 56% (1.22 g); Appearance = yellowish honey-like material; ^1^H NMR (500 MHz, Acetone-*d*_6_) δ 3.23 (t, *J* = 5.0 Hz, ^24^H), 3.35 (d, *J* = 11.8 Hz, ^49^H), 3.48–3.55 (m, ^92^H), 3.68 (dt, *J* = 12.1, 4.9 Hz, ^36^H), 3.93–3.99 (m, ^24^H), 4.38 (q, *J* = 5.7 Hz, ^12^H), 6.73–6.78 (m, ^24^H), 7.19 (d, *J* = 8.1 Hz, ^24^H), 7.70 (s, ^6^H); ^13^C NMR (126 MHz, Acetone-*d*_6_) 47.26, 49.78, 50.51, 56.28, 67.41, 68.49, 69.33, 69.56, 69.85, 70.20, 70.33, 70.46, 70.57, 78.19, 78.47, 114.24, 123.68, 129.99, 130.00, 131.52, 144.05, 144.11, 158.08; LC/MS: exact mass calculated for [M]^+^, C_204_H_290_N_60_O_48_, is 4348.2096 amu; found as charge states, [M + 3H]^2+^ ~ [M + 6H]^6+^, which were deconvoluted to 4348.2090 amu.

#### 4.7.9. Compound **8** (G2)

Compound **7** (0.35 g, 4348.21 g/mol, 0.080 mmol) and compound **3b** (0.47 g, 387.43 g/mol, 1.21 mmol) were weighed into a 35 mL microwave reaction vessel. Then, copper granules (500 mg) and a stir bar were added. Anhydrous THF was added to the vessel via a cannula needle, and the reaction mixture was purged with argon. The mixture was stirred until all reactants were fully dissolved. The microwave reaction was run for 15 h at 77 °C and 150 W under N_2_ gas. The reaction was completed within this time. The mixture was filtered through Celite 545 with THF. The filtrate was treated with saturated EDTA solution for 1 h and dried on a rotary evaporator. The dried mixture was dissolved in chloroform and washed with water three times. The organic layer was dried over MgSO_4_. After filtering out MgSO_4_, the filtrate was evaporated and then loaded onto a Sephadex LH20 column for purification. Purification was performed using CHCl_3_-MeOH (90:10) to separate the target compound from leftover starting materials, any remaining EDTA, and the EDTA-Cu complex. The target compound was eluted first. Fractions containing the pure target compound were combined based on HPLC and LC/MS results, dried, and kept under vacuum until NMR analysis.

Yield = 82% (0.59 g); Appearance; yellowish sticky solid; ^1^H NMR (500 MHz, CDCl_3_) δ 3.41 (s, ^24^H), 3.43–3.48 (m, ^26^H), 3.50 (s, ^48^H), 3.53–3.58 (m, ^54^H), 3.64 (s, ^12^H), 3.66–3.69 (m, ^24^H), 3.73 (s, ^24^H), 3.80 (s, ^180^H), 3.95 (dt, *J* = 6.3, 3.2 Hz, ^24^H), 4.40 (q, *J* = 6.1 Hz, ^12^H), 4.46 (t, *J* = 5.1 Hz, ^24^H), 5.81 (s, ^4^H), 6.60 (s, ^48^H), 6.73 (dd, *J* = 8.3, 2.6 Hz, ^24^H), 7.15–7.21 (m, ^24^H), 7.53 (s, ^5^H), 7.54 (s, ^2^H), 7.55 (s, ^11^H); ^13^C NMR (126 MHz, CDCl_3_) δ 47.56, 47.78, 49.96, 50.12, 56.25, 56.39, 57.52, 67.29, 68.68, 69.42, 69.54, 69.71, 70.34, 70.50, 70.56, 71.62, 77.37, 105.54, 114.27, 114.37, 123.42, 123.74, 129.99, 130.12, 131.35, 131.38, 133.73, 144.76, 147.03, 157.70; LC/MS: theoretical (monoisotopic) mass calculated for target, C_456_H_590_N_72_O_120_, is 8994.2279 amu; found as charged states of [M + 4H]^4+^ ~ [M + 11H]^11+^; deconvoluted to 8994.1968 amu.

## 5. Conclusions

Two syringaldehyde-based antioxidant dendrimers were built on a scaffold derived from D-mannitol. The G1 dendrimer contains 12 syringic units, while G2 carries 24 units. The antioxidant dendrimers reported in this study are among the largest antioxidant dendrimers known to function as antioxidants on their own. Both G1 and G2 exhibit significantly higher DPPH radical scavenging activities compared to the syringaldehyde starting material and BHT. Overall, G2 is about twice as effective as G1 in neutralizing DPPH radicals. Additionally, the dendrimers protect β-carotene much more effectively than the controls. These results suggest that the presence of multiple phenolic units with the correct structural requirements, such as EDG at *ortho* and/or *para* positions, results in strong antioxidant activity. Strategically assembling weak antioxidants into dendritic structures can further enhance their antioxidant activities through cooperative dendritic effects. G1 led to a decrease in cell viability at ≥32 μM, while G2 caused a gradual decrease starting at 0.1 μM. This indicates that the larger antioxidant dendrimer is more cytotoxic than the smaller one and requires further improvement. We are currently evaluating these compounds in various cell lines to assess their overall cytotoxicity and anti-inflammatory effects, and to explore their potential applications in relevant fields.

## Figures and Tables

**Table 1:** IC50 values for antioxidants determined in the DPPH assay.

Antioxidant	IC_50_ (µM)
Compound **8** (G2)	0.7
Compound **5** (G1)	1.36
Syringaldehyde	260
BHT	880

## Data Availability

The original contributions presented in this study are included in the article/Supplementary Material. Further inquiries can be directed to the corresponding author.

## Supplementary Materials

The following supporting information can be downloaded at: https://www.mdpi.com/article/10.3390/ijms262210966/s1.
