# Correction: Agbemade et al. Synthesis and Evaluation of Powerful Antioxidant Dendrimers Derived from D-Mannitol and Syringaldehyde. *Int. J. Mol. Sci.* 2025, *26*, 10966

**DOI:** 10.3390/ijms27114664

**Published:** 2026-05-22

**Authors:** Blessed Agbemade, Amanda R. Clark, Cyprien N. Nanah, Fati Haruna, Aundrea E. Stengard, Skylar A. Medes, Ashlyn M. Lapratt, Samara L. Morehouse, Rebecca L. Uzarski, Choon Young Lee

**Affiliations:** 1 Department of Chemistry and Biochemistry, Central Michigan University, Mount Pleasant, MI 48859, USA; agbem1b@cmich.edu (B.A.); clark3ar@cmich.edu (A.R.C.); nanah1cn@cmich.edu (C.N.N.); harun1f@cmich.edu (F.H.); steng1ae@cmich.edu (A.E.S.); medes1sa@cmich.edu (S.A.M.); lapra1am@cmich.edu (A.M.L.); moreh1sl@cmich.edu (S.L.M.); 2 Science of Advanced Materials Program, Central Michigan University, Mount Pleasant, MI 48859, USA; 3 Department of Biology, Central Michigan University, Mount Pleasant, MI 48859, USA; uzars2rl@cmich.edu

There was an error in the original publication [1]. In the Abstract section, Results, Discussion, and the Materials and Methods section.

## 1. Error in Figure

Scheme 2 in the 2nd paragraph of the Results section should be replaced with the new Scheme 2 shown below because the current version shows the percent yield for only one compound when there are two compounds in the scheme. The authors state that the scientific conclusions are unaffected.

## 2. Text Correction

**1.**  **Abstract**

There is a typo in the Abstract of the original manuscript: the number 1251 should be 1257. This is simply a typo. The exact same information was provided under 2.2. DPPH Assay Results in the Results section. The corrected sentence is “They are also 1257- and 647-times more effective than butylated hydroxytoluene (BHT) (880 μM).”

**2.**  **Section 2. Results**

In the 1st sentence in the 3rd paragraph, the authors wish to add the Scheme number at the end of the sentence so that the readers can be directed to the Scheme that contains the structure that is being explained in the sentence. The revised sentence: “To construct dendrimers, D-mannitol, a six-carbon chain with each carbon atom bonded to an OH group, served as the core (Scheme 3).” The legend for Scheme 3 should revise to “Scheme 3. Synthesis of the G1 target dendrimer. Note: the same numbers with the same color across the two chains show identical chemical shifts in the NMR spectra.”

The 2nd sentence of the 3rd paragraph, “Its internal OH groups have R configurations, orienting all six OH groups in different directions, which helps reduce steric hindrance in subsequent reactions involving these OH groups”, was modified because this sentence does not explain how many internal OH groups are present. Although the readers can count themselves, the authors would like to be clearer by including the number of internal OH groups. We then divided the sentence into two parts to improve flow and make the text easier to understand. The main message was not altered. The revised sentence is “Its four internal OH groups have R configurations, causing all six OH groups to orient in different directions. This arrangement helps reduce steric hindrance in subsequent reactions involving these groups.”

In the 4th paragraph, the sentence “Compound **4** was observed as [M + H]^+^ and [M + NH_4_]^+^ with average masses of 861.4431 amu and 878.4692 amu, respectively; their calculated masses are 861.4398 amu and 878.4669 amu, respectively” was modified. When we compared the explanation in the original manuscript with the data sheet provided in the Supplementary Materials, we observed that more than two ionized species were present. Please note that these are all target compounds with different ions attached, as is common for any sample and any compound. The same compound can be ionized by different cations. They are all deconvoluted to the same target compound. We are just reporting that our target compound was observed with four different ions attached. To match the text with the experimental data sheet, we added the other two ions in the text. We replaced the term “average” with “monoisotopic” to be more accurate. For calculated masses, depending on the software used, the values vary slightly. We used ChemDraw Pro 12.0 to calculate all theoretical masses for consistency. Then, the sentence was divided into two to improve readability. None of these is critical information. The authors do not object to leaving the information unchanged; however, the change would be preferable because the description aligns with the experimental data. The revised sentence is “Compound **4** was detected as [M + H]^+^, [M + NH_4_]^+^, [M + Na]^+^, and [M + K]^+^ ions, exhibiting monoisotopic masses of 861.4431, 878.4692, 883.4243, and 899.3969 amu, respectively. The theoretical monoisotopic masses for these adducts are 861.4398, 878.4663, 883.4217, and 899.3957 amu, respectively.”

In the same paragraph, there is a typo for the version of MestreNova NMRPredict, which was changed from 16.1 to 16.0. The corrected sentence is “Our assignments were compared with the chemical shifts and integration values estimated by a program (MestreNova NMRPredict version 16.0)”.

**3.**  **Section 3. Discussion**

In the 5th paragraph, the authors revised the following sentences to align with the subsequent sections. “Nevertheless, size-dependent cytotoxicity should be a primary consideration in the design of nano-antioxidants, as excessive toxicity can reduce their viability for various applications. It is important to optimize the size to minimize cytotoxicity while maintaining antioxidant activity. Based on the literature, size is just one factor that influences cytotoxicity; particle type, shape, and surface properties also play a role [64]. The same particle can have different toxic effects depending on the cell type [63]. Another factor contributing to cytotoxicity, which may be related to our dendrimers, is hydrophobicity [68–70]. The increased cytotoxicity of G2 may result from a higher number of hydrophobic phenolic units on its surface compared to G1.”

The term “size-dependent” was removed because other factors causing cytotoxicity are mentioned in subsequent sentences. We are focusing on reducing not only size-dependent cytotoxicity but also cytotoxicity from other factors. Please note that all other changes in this paragraph are intended to improve readability and do not alter the original message.

The corrected version is “Nevertheless, minimizing cytotoxicity should be a primary consideration in the design of nano-antioxidants, as excessive toxicity can limit their viability for various applications. It is crucial to optimize size to reduce cytotoxicity while maintaining antioxidant activity. Based on the literature, size is just one factor that influences cytotoxicity; particle type, shape, and surface properties also play significant roles [64]. Additionally, the same particle can exhibit different toxic effects depending on the specific cell type involved [63]. Another contributing factor to cytotoxicity, which may be relevant to our dendrimers, is hydrophobicity [68,69,70]. The increased cytotoxicity of G2 may be attributed to its higher number of hydrophobic phenolic units on the surface compared to G1.”

**4.**  **Section 4. Materials and Methods**

- Section 4.3. Characterization

At the end of the 1st paragraph, there is a sentence with an error: We originally intended to submit our manuscript as a Letter, which is why we placed all experimental methods and supporting data in the Supplementary Materials. After the journal office requested a full manuscript instead, we restructured our submission by moving all experimental methods into the main text and retaining only the supporting data in the Supplementary Materials. Unfortunately, we overlooked updating this statement to reflect these changes.

The corrected sentence is “^1^H NMR and ^13^C NMR spectra for each compound are shown in Supplementary Materials.”

- Section 4.6. Cell Viability Assay

There is a typo in the main text, where it says 24 h, and the experimental method section says 18 h. The incubation period is 24 h rather than 18 h.

The corrected sentence is “To assess viability, 100 μL of cells (5 × 10^5^/mL) were added to 96-well plates and incubated with 100 μL media control or test compound at varying concentrations for 24 h.”

- Section 4.7.2. Compound **2b**

Although not essential, the authors converted all quantities from mol to mmol to ensure consistency with all the other method sections. The corrected sentence: “2-[2-(2-Chloroethoxy)ethoxy]ethanol (10 mL, d = 1.16 g/mL, MW = 168.62 g/mol, 68.79 mmol) and sodium azide (4.9195 g, 65.01 g/mol, 75.67 mmol) were dissolved in DMF (200 mL).”

The unit “mol” in the 6th sentence was also changed to mmol. Additionally, we noticed that the molecular weight of compound **1b** was incorrect. Therefore, we changed it. The corrected version: “The dried material (compound **1b**, 13.96 g, 175.10 g/mol, 79.73 mmol) in the round-bottom flask was transferred to a 500 mL three-neck round-bottom flask cooled in a dry-ice bath containing dry ice in isopropanol.”

In the 8th sentence, the corrected version: “Then, triethylamine (18 mL, 0.726 g/mL, 101.06 g/mol, 129.31 mmol) was added to the stirring solution.”

- Section 4.7.3. Compound **3a** (Internal BB)

In the 1st sentence, the quantity of propargyl amine was repeated, and in addition, the quantity is incorrect. Upon review of the note, the corrected quantity is 1.2 mL, not 1 mL, corresponding to 18.89 mmol.

The corrected sentence: “4-Hydroxybenzaldehyde (2.30 g, 122.12 g/mol, 18.83 mmol) was dissolved in methanol, and then propargylamine (1.2 mL, 0.867 g/mL, 55.08 g/mol, 18.89 mmol) was added.”

- Section 4.7.4. Compound **3b** (Surface BB)

There is an error in the mass reported in the reaction’s percent yield. One of our undergraduate students focuses exclusively on synthesizing BBs. She regularly produces the same compound for the graduate students. At times, she records the purified product weight in grams in her notebook, while at other times, she notes the % yield. Typically, the BB synthesis reactions yield between 80% and 90%. However, using the numbers in the original manuscript, the percent yield is 106%, which is not possible. Therefore, we reviewed her notebook, which indicates a yield of 86%, meaning the mass should be 5.24 g, not 6.49 g.

The corrected sentence: “Yield = 86% (5.24 g)”

- Section 4.7.5. Compound **4** (G0.5)

At the bottom of the second paragraph, where LC/MS data are listed, we added two additional ionized species to align with the data sheet in the Supplementary Materials; this is already explained above. This is just experimental data for the information described in the 4th paragraph of the Results section. For calculated mass, slight variations may occur depending on the software used. We sometimes use ACD/ChemSketch and other times ChemDraw. To ensure consistency, we used ChemDraw and recalculated the theoretical masses of the four charged ions. Please note that these theoretical values are to be compared with the observed values. In addition, adding two additional ionized species does not alter the scientific conclusions. All of them are deconvoluted to the desired target compound.

The corrected version is “LC/ESI-TOF MS: exact *m*/*z* calculated for [M + H]^+^, C_30_H_57_N_18_O_12_, is 861.4398 *m*/*z*, found as 861.4431, *m*/*z* calculated for [M + NH_4_]^+^, C_30_H_60_N_19_O_12_, is 878.4663 amu, found as 878.4692 amu, and *m*/*z* calculated for [M + K]^+^, C_30_H_56_N_18_O_12_K, is 899.3957 amu, found as 899.3969 amu.”

- Section 4.7.9. Compound **8** (G2)

This sentence contains a small typo and should be corrected to “Compound **7** (0.35 g, 4348.21 g/mol, 0.080 mmol) and compound **3b** (0.47 g, 387.43 g/mol, 1.21 mmol) were weighed into a 35 mL microwave reaction vessel.”

The authors state that the scientific conclusions are unaffected. This correction was approved by the Academic Editor. The original publication has also been updated.

## Figures and Tables

**Scheme 2:**
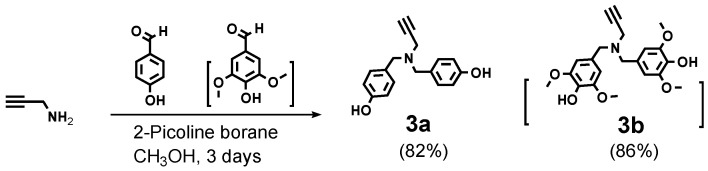
Synthesis of building blocks.
