# Supplementary material for: Synthesis and Evaluation of Powerful Antioxidant Dendrimers Derived from D-Mannitol and Syringaldehyde
Source: Int J Mol Sci. 2025 Nov 12;26(22):10966. doi: 10.3390/ijms262210966 (PMC12652216; doi:10.3390/ijms262210966)
Supplement: Supplementary file 1 [file ijms-26-10966-s001.zip › ijms-3939454-supplementary highlight.pdf]

Supporting Information

Synthesis and Evaluation of Powerful Antioxidant Dendrimers Derived from D-Mannitol and Syringaldehyde

Blessed Agbemade,[1,2] Amanda Clark,[1] Cyprien N. Nanah,[1] Fati Haruna,[1] Aundrea Stengard,[1] Skylar Medes,[1] Ashlyn Lapratt,[1] Samara L. Morehouse,[1] Rebecca L. Uzarski,[3] and Choon Young Lee*[,1,2]

1. Department of Chemistry and Biochemistry, Central Michigan University, Mount Pleasant, MI 48859, USA
2. Science of Advanced Materials Program, Central Michigan University, Mount Pleasant, MI 48859, USA
3. Department of Biology, Central Michigan University, Mount Pleasant, MI 48859, USA

E-mail address: agbem1b@cmich.edu, clark3ar@cmich.edu, nanah1cn@cmich.edu, harun1f@cmich.edu, steng1ae@cmich.edu, medes1sa@cmich.edu, lapra1am@cmich.edu, moreh1sl@cmich.edu, uzars2rl@cmich.edu,

Correspondence: lee1cy@cmich.edu

Supplementary Figures

Each NMR spectrum contains the compound structure with all H and C atoms numbered to help assign NMR signals. However, not all atoms were numbered because the D-mannitol-based dendrimers acted as if there was a symmetry in terms of chemical shifts. Therefore, it should be noted that the atoms, which were not numbered, have the same chemical shifts as the same functional groups that are equal distances away from the D-mannitol core. The atoms numbered are representatives of functional groups within the entire molecule.

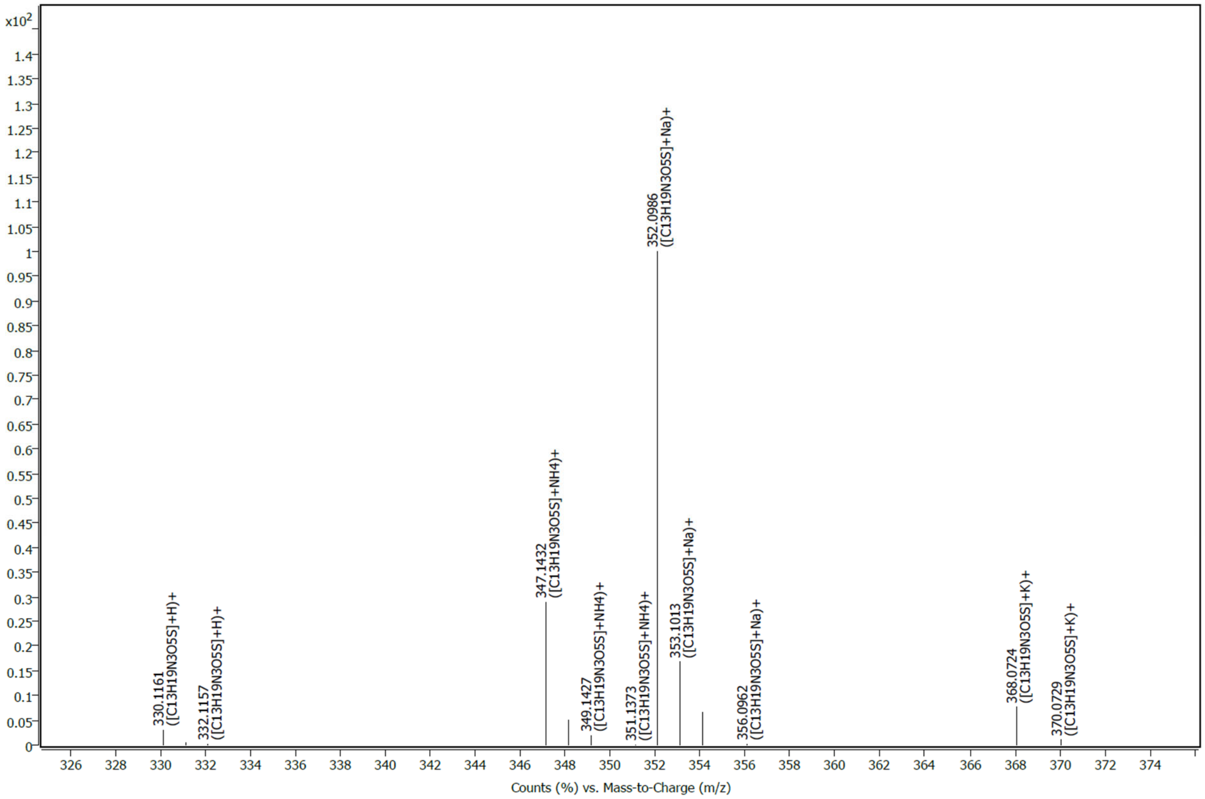

Figure S1. MS spectrum of compound **2b**.

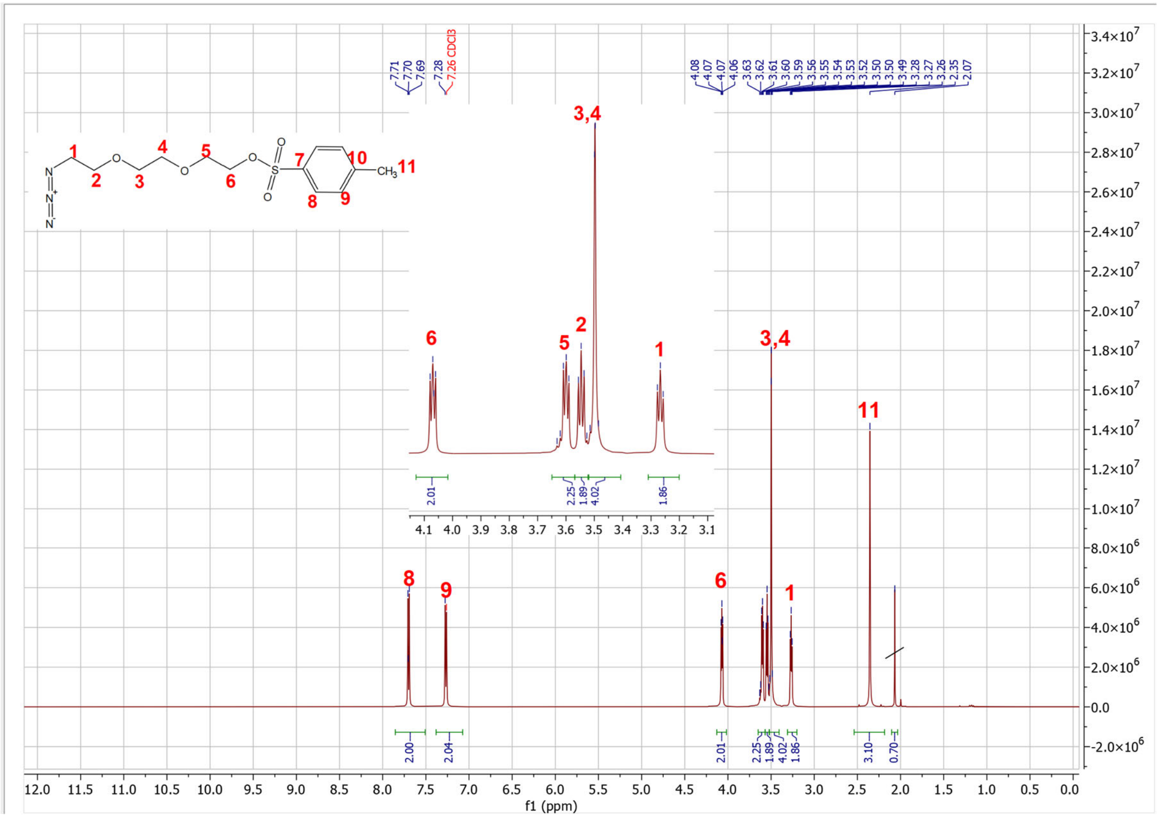

Figure S2. ^{1}H NMR of compound **2b**.

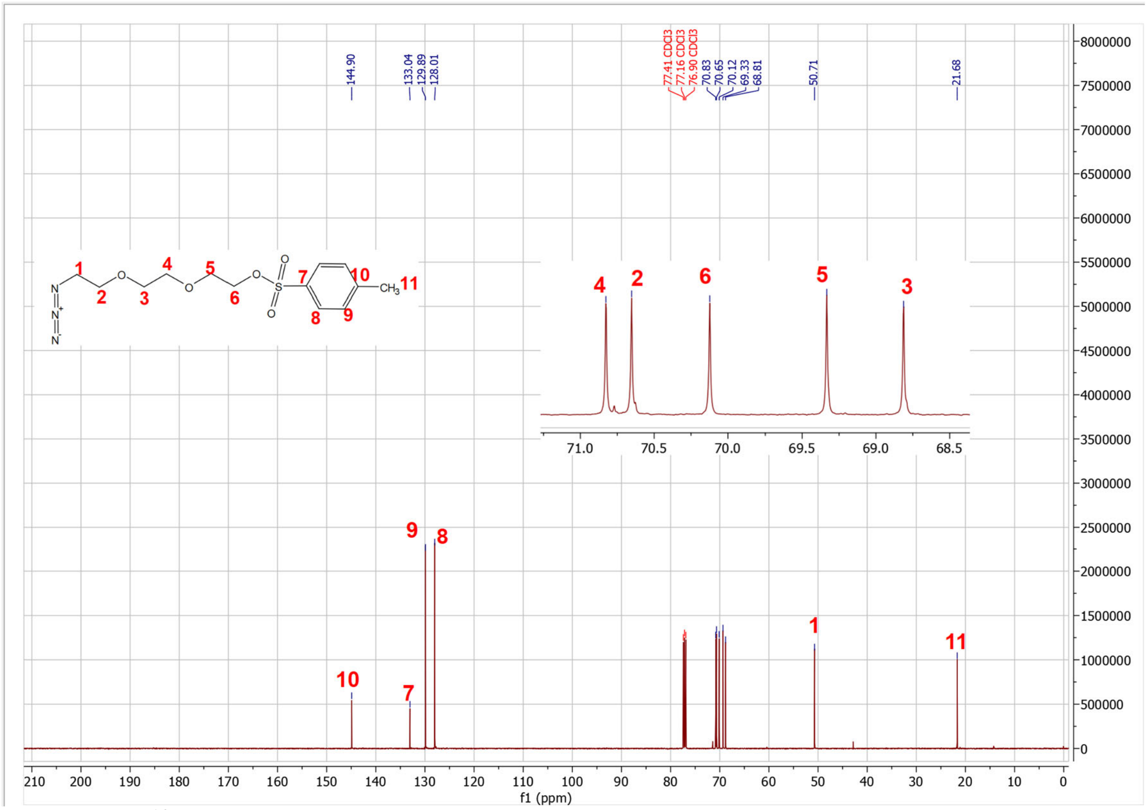

Figure S3. ^{13}C NMR of compound **2b**.

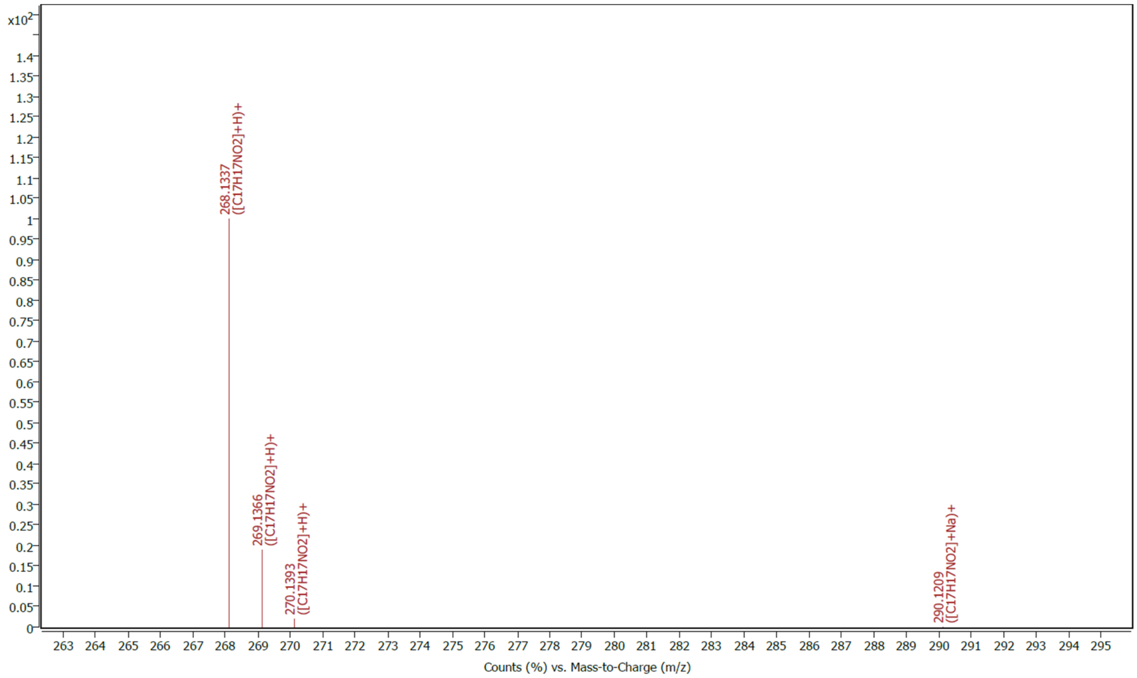

Figure S4. MS spectrum results of compound **3a**.

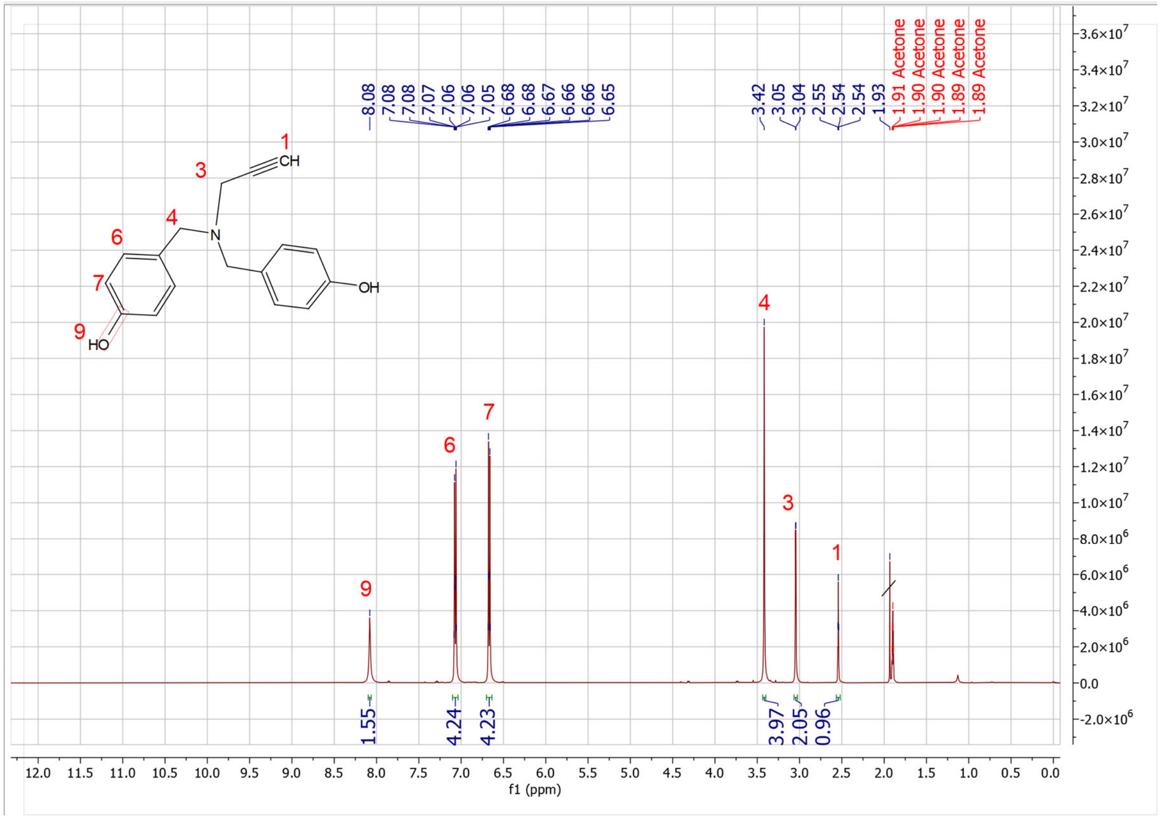

Figure S5. ¹H-NMR of compound **3a**.

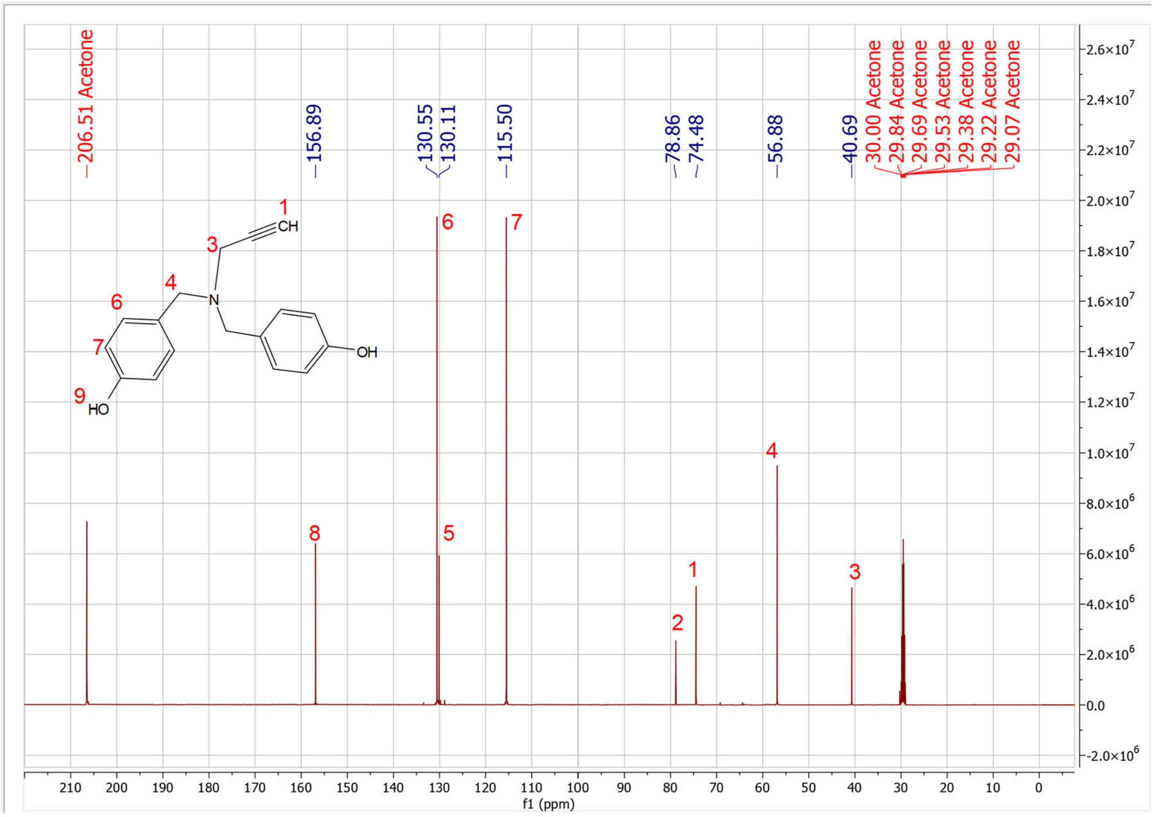

Figure S6. ^{13}C-NMR of compound **3a**.

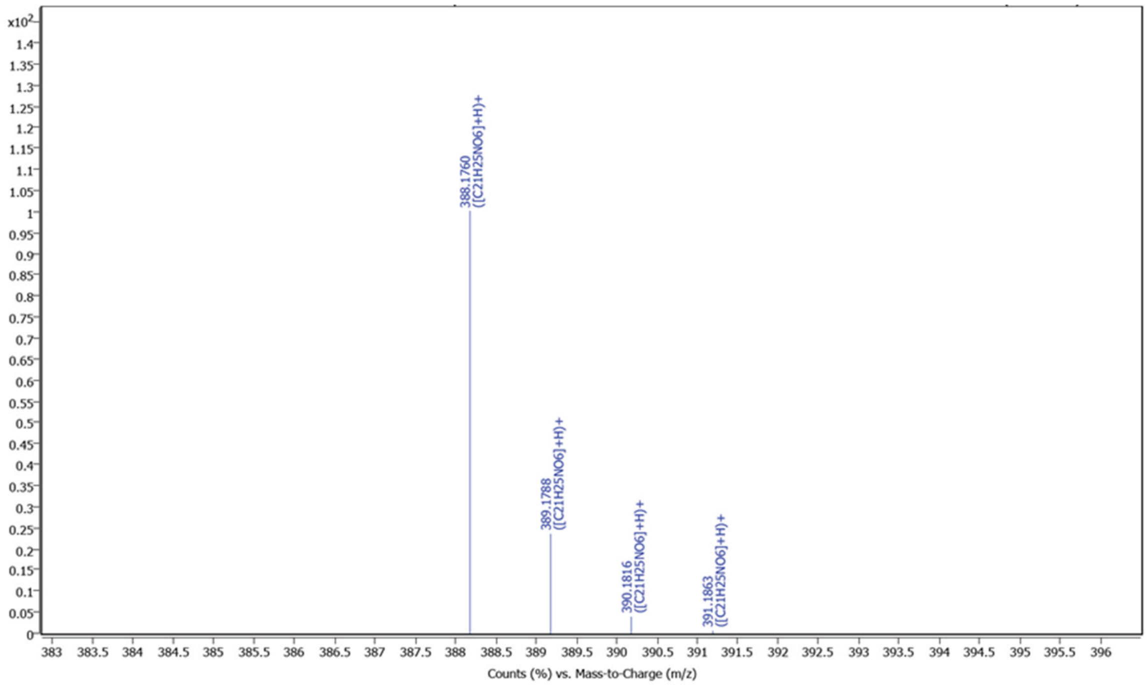

Figure S7. MS spectrum results of compound **3b**.

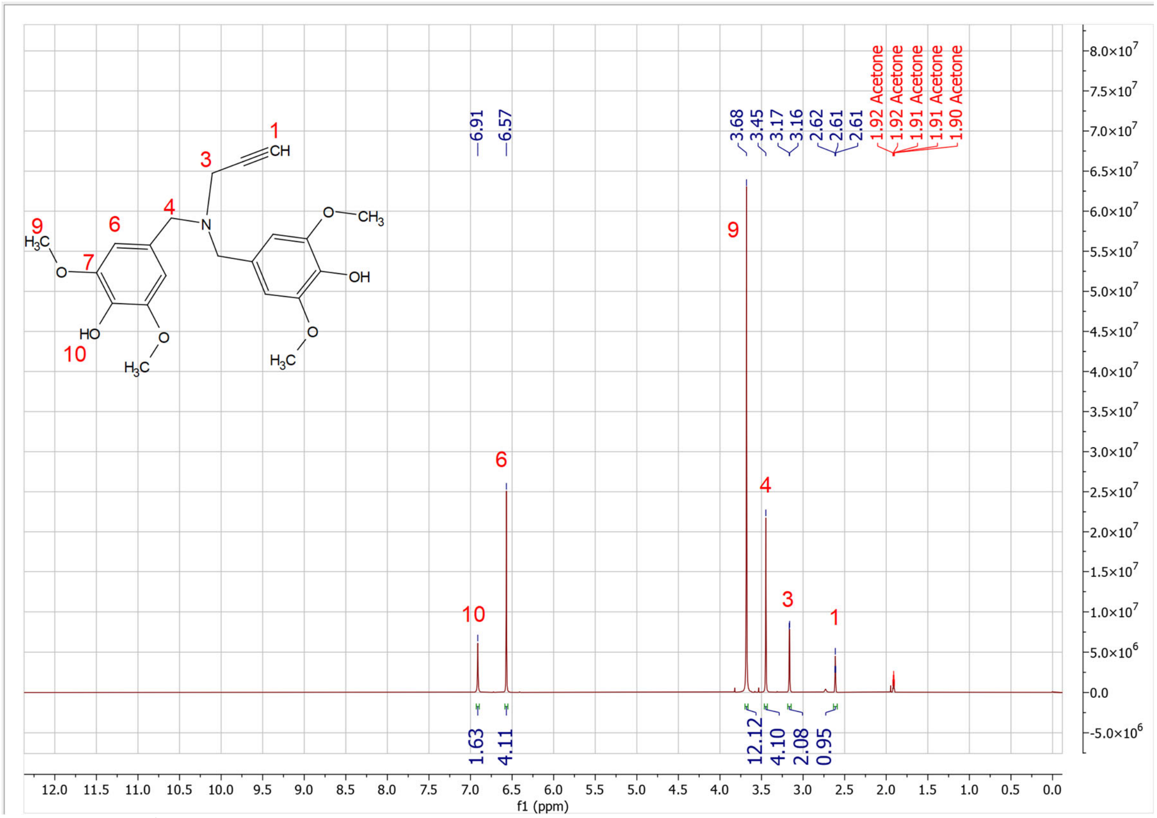

Figure S8. ^{1}H-NMR of compound **3b**.

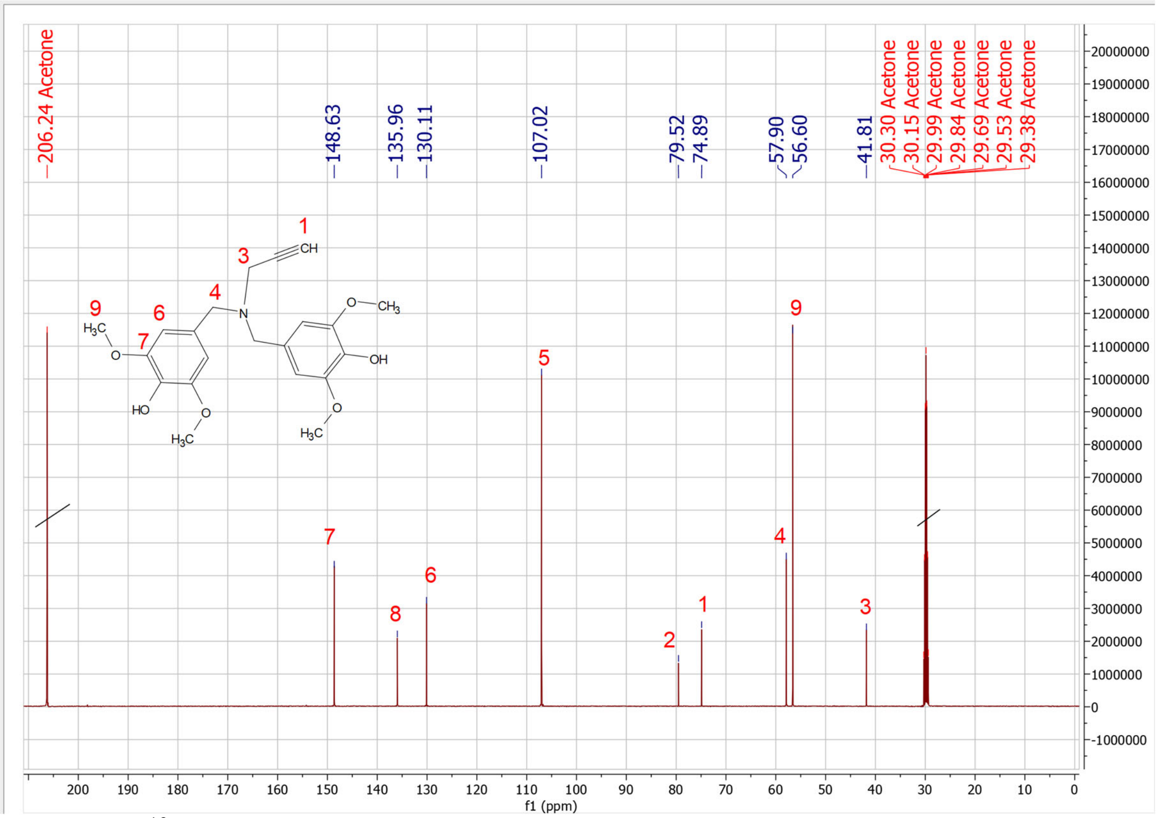

Figure S9. ^{13}C-NMR of compound **3b**.

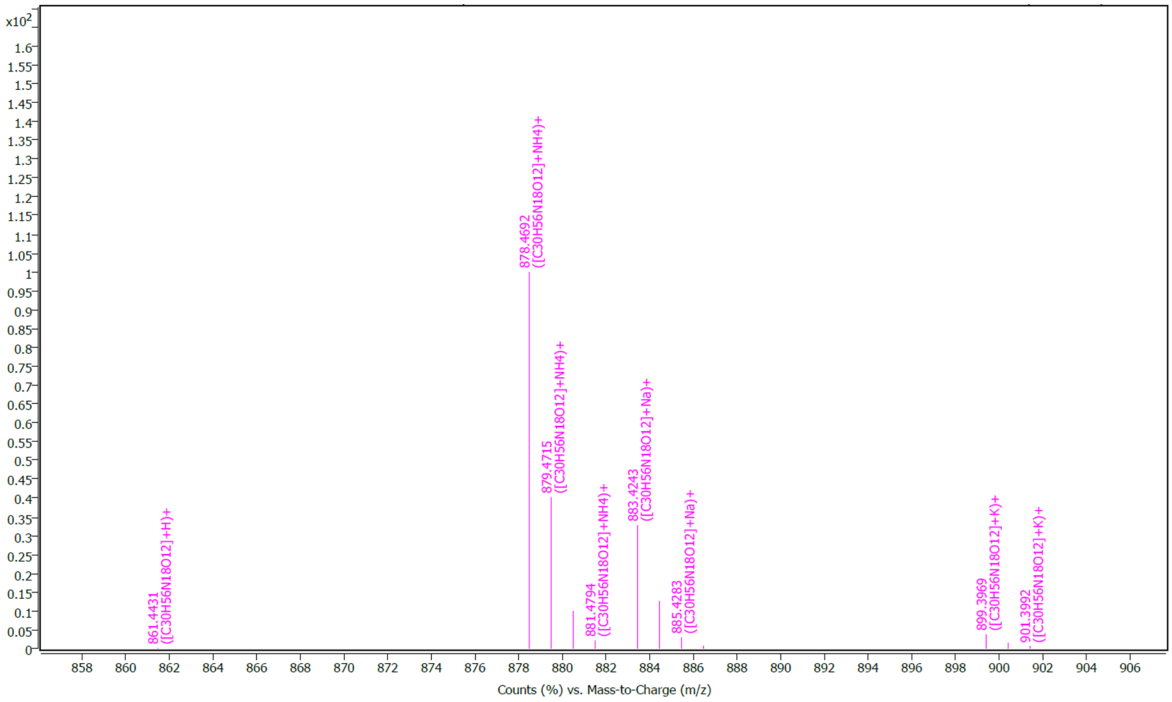

Figure S10. MS spectrum of compound **4**.

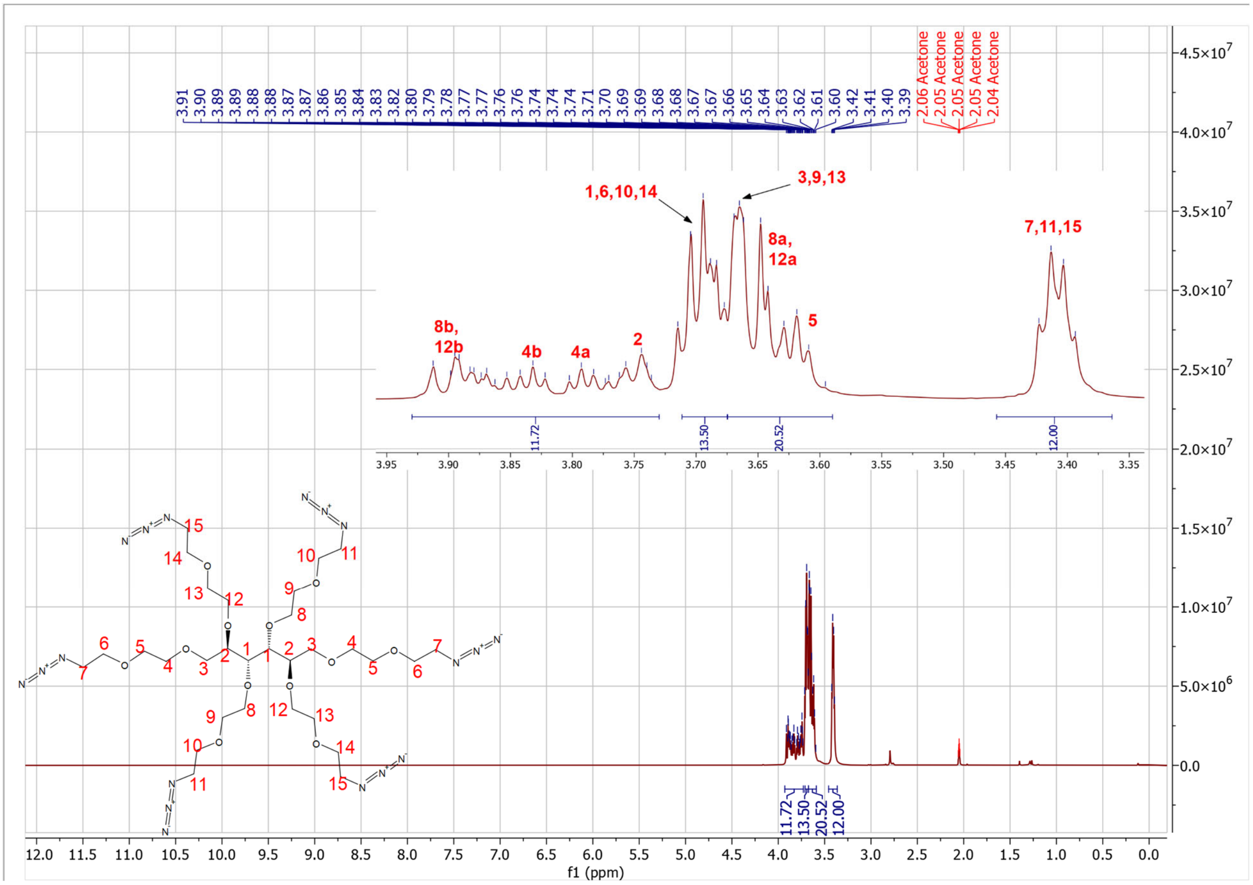

Figure S11. ^{1}H NMR spectrum of compound **4**.

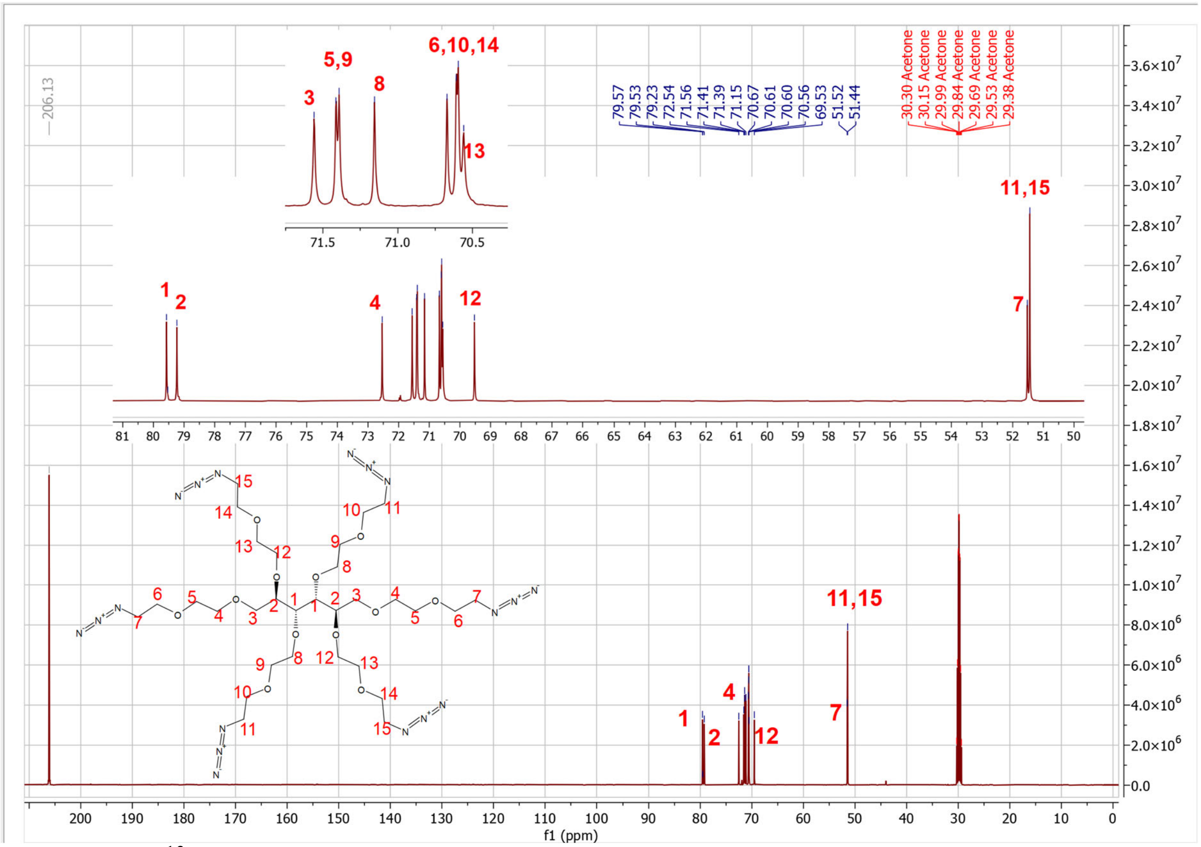

Figure S12. ^{13}C NMR spectrum of compound **4**.

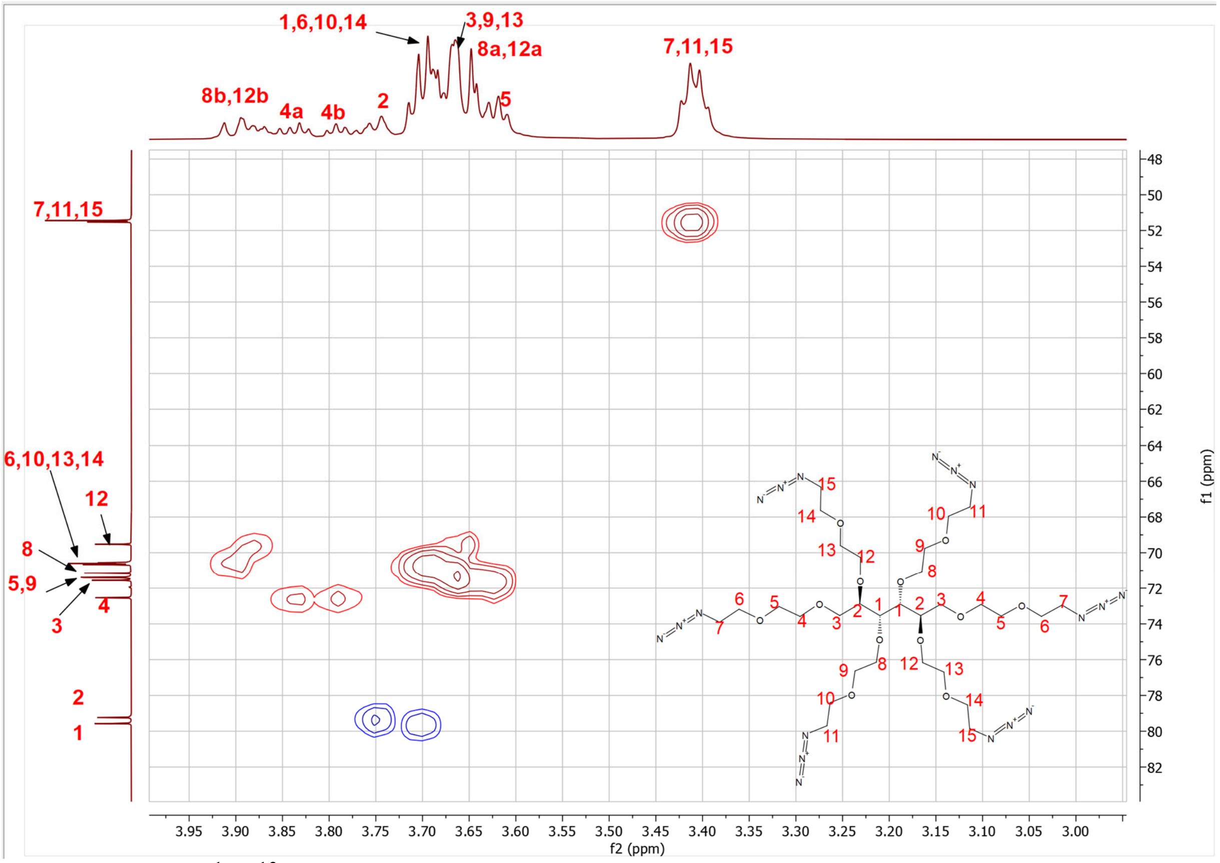

Figure S13. 2D ^{1}H-^{13}C HSQC NMR spectrum of compound **4**.

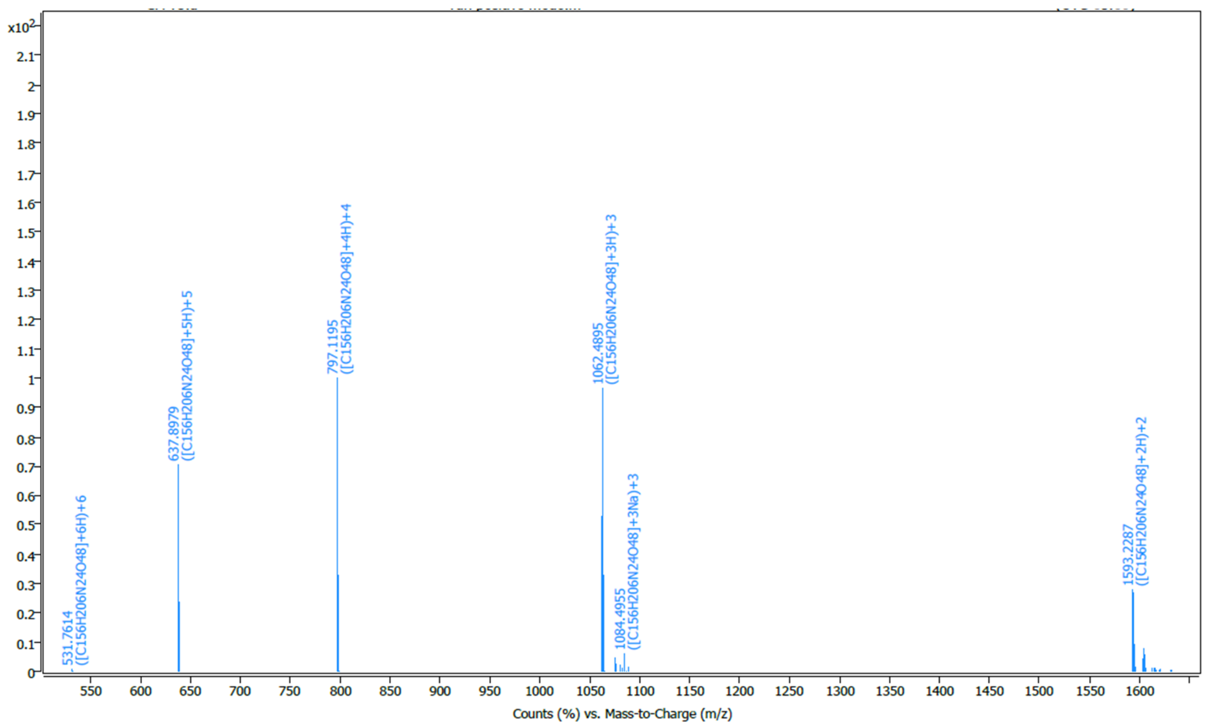

Figure S14. MS spectrum of compound **5**.

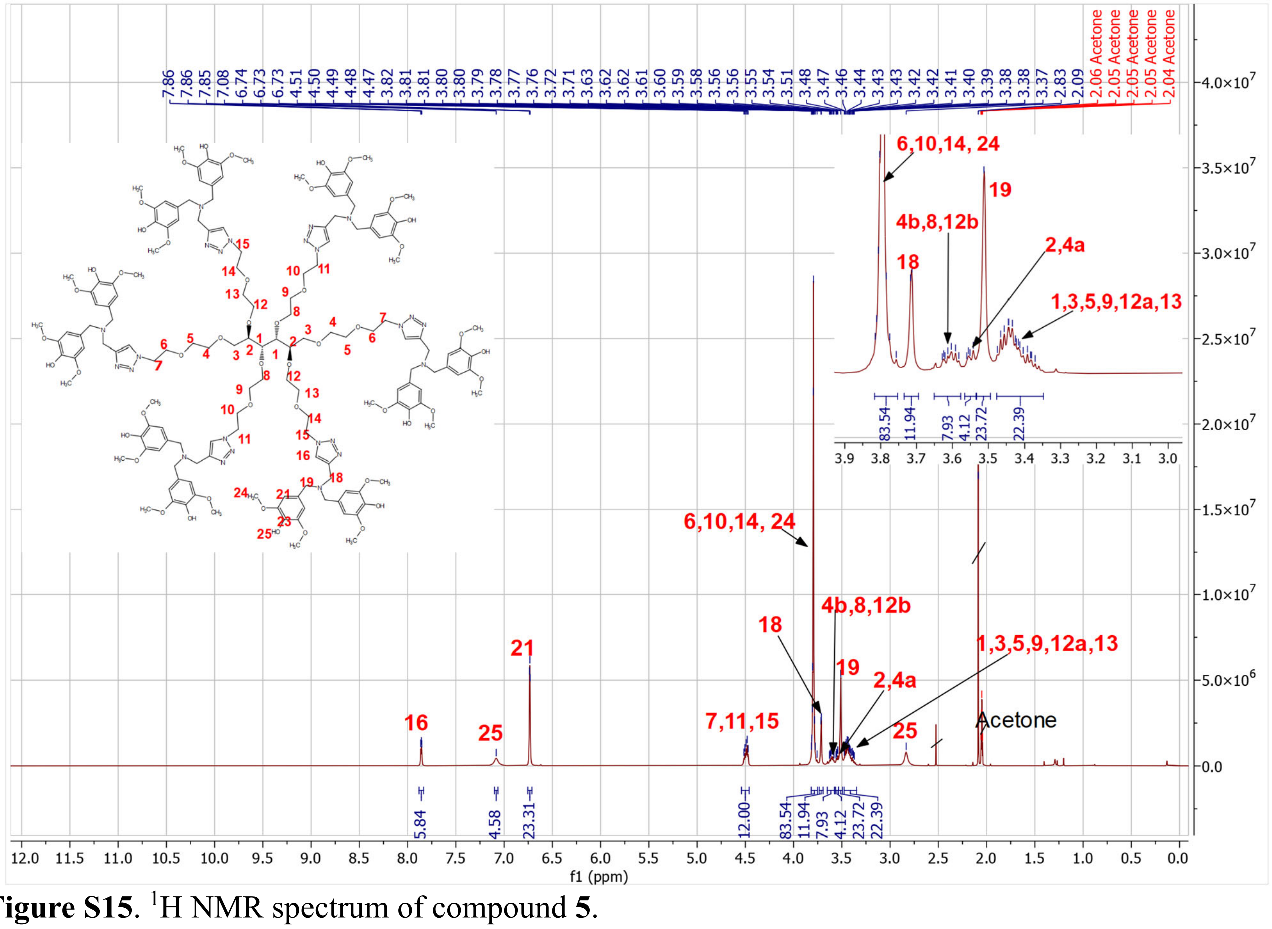

Figure S15. ^{1}H NMR spectrum of compound **5**.

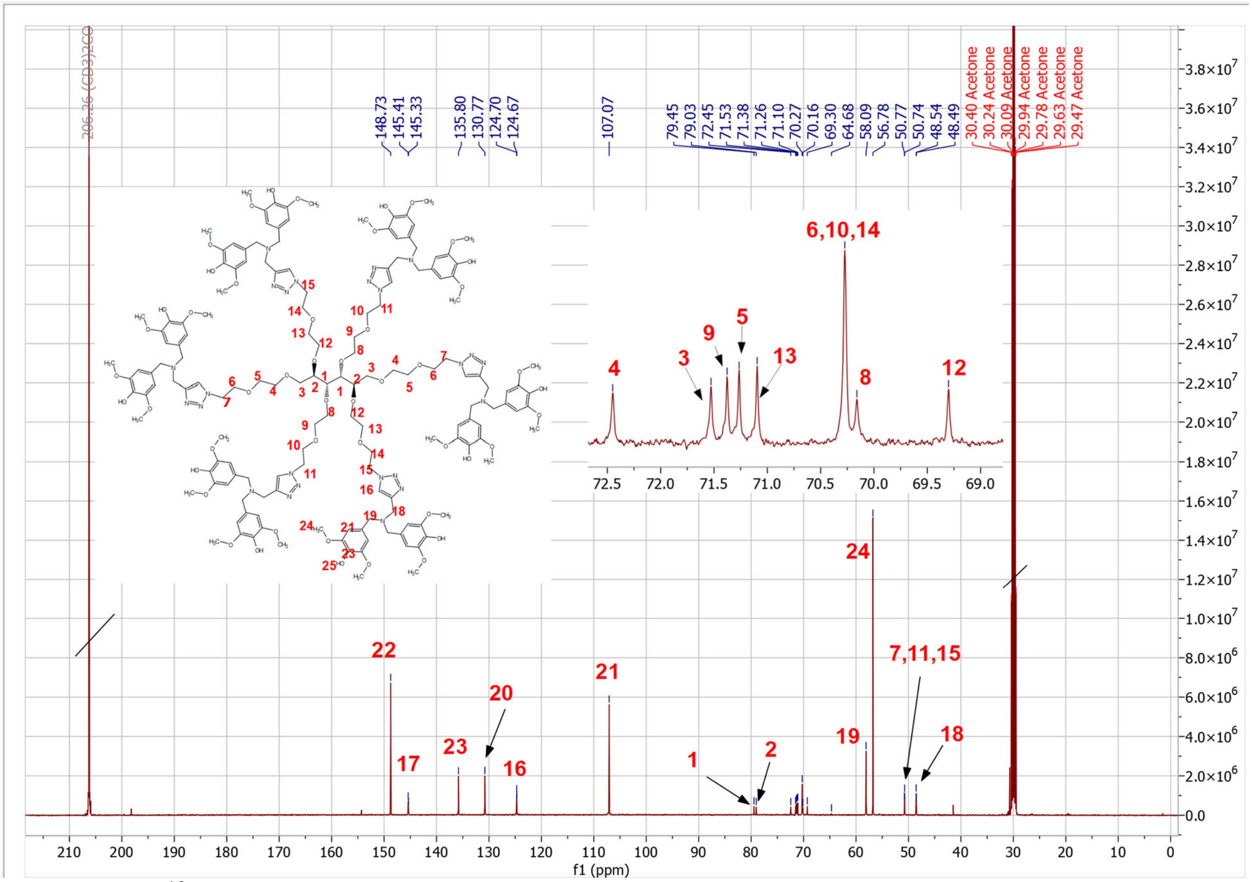

Figure S16. ^{13}C NMR spectrum of compound **5**.

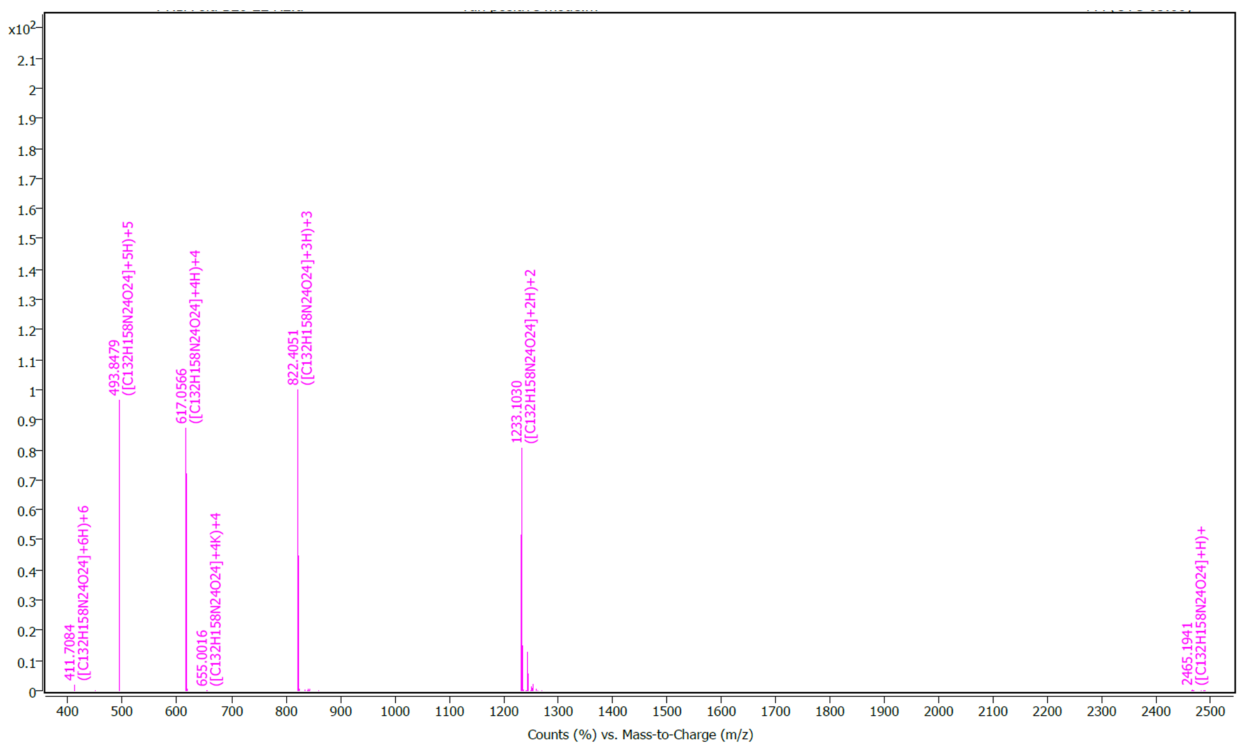

Figure S17. MS spectrum of compound **6**.

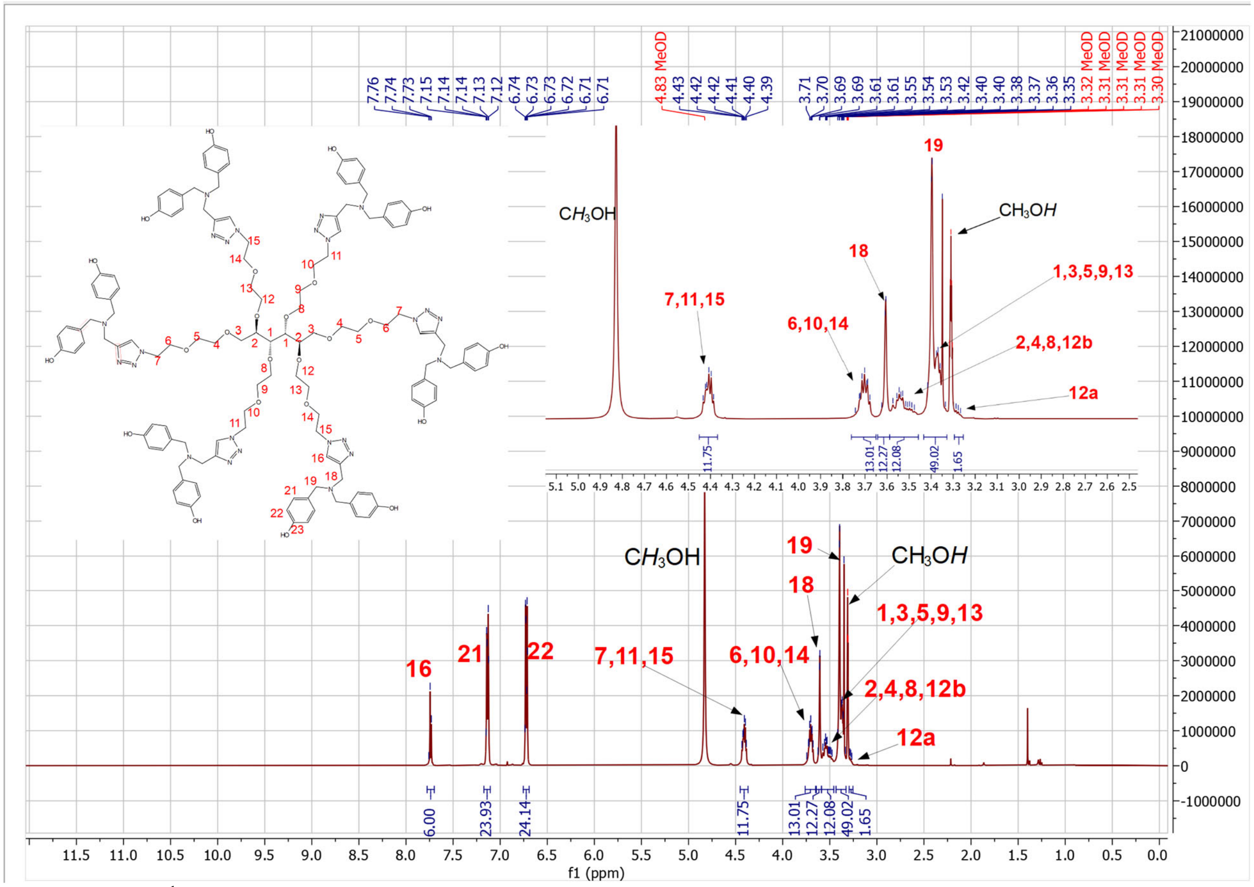

Figure S18. ^{1}H NMR spectrum of compound **6**.

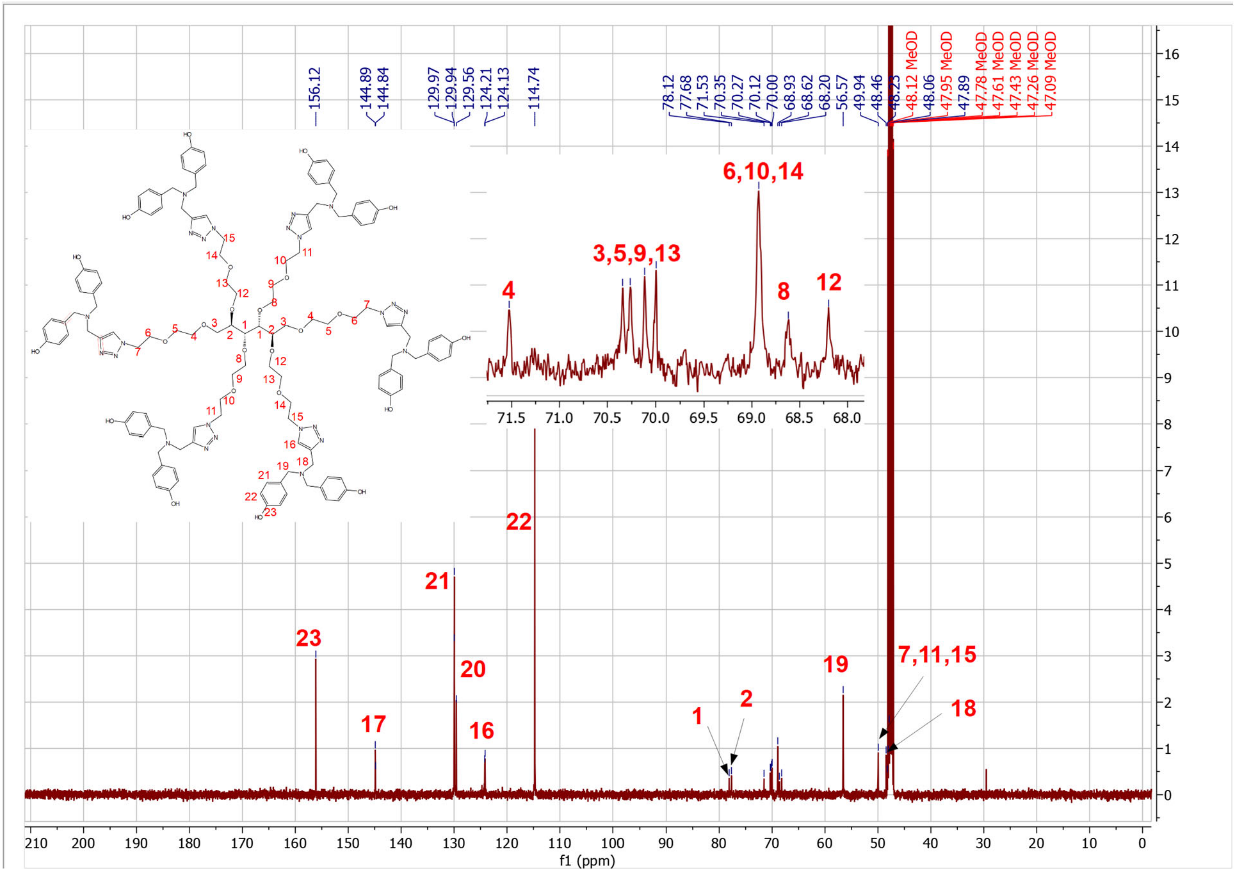

Figure S19. ^{13}C NMR spectrum of compound **6**.

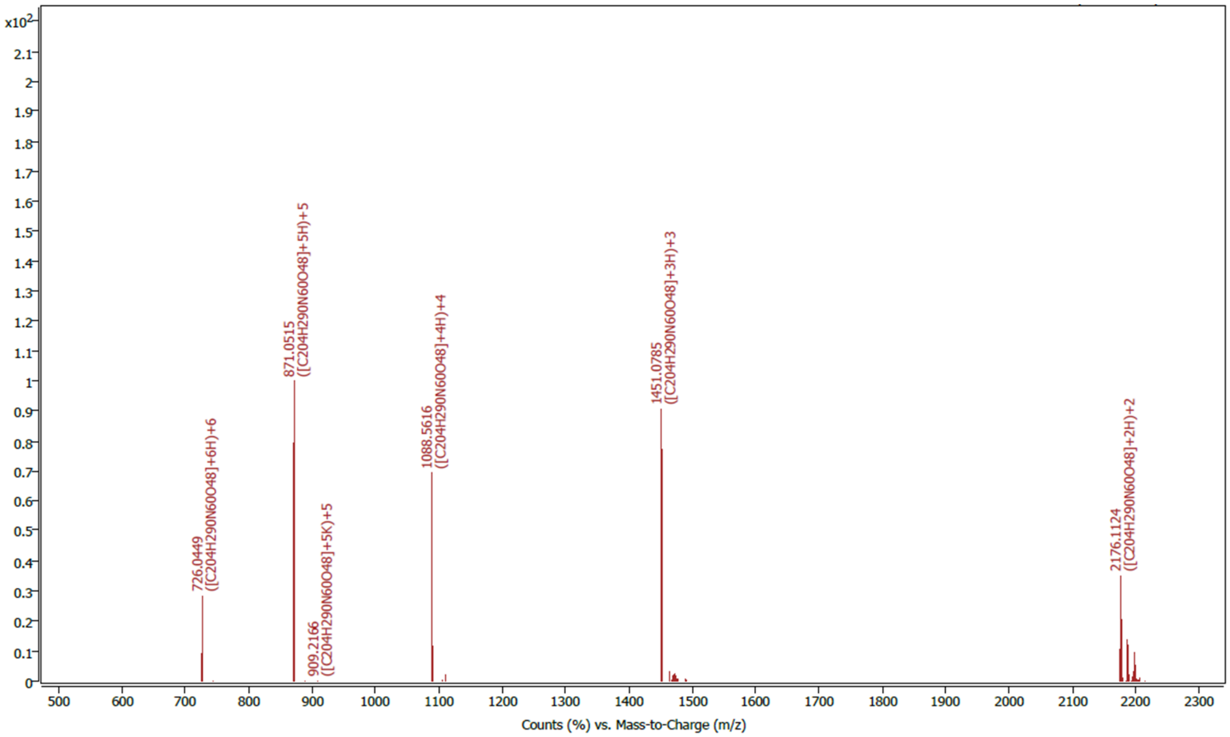

Figure S20. MS spectrum of compound **7**.

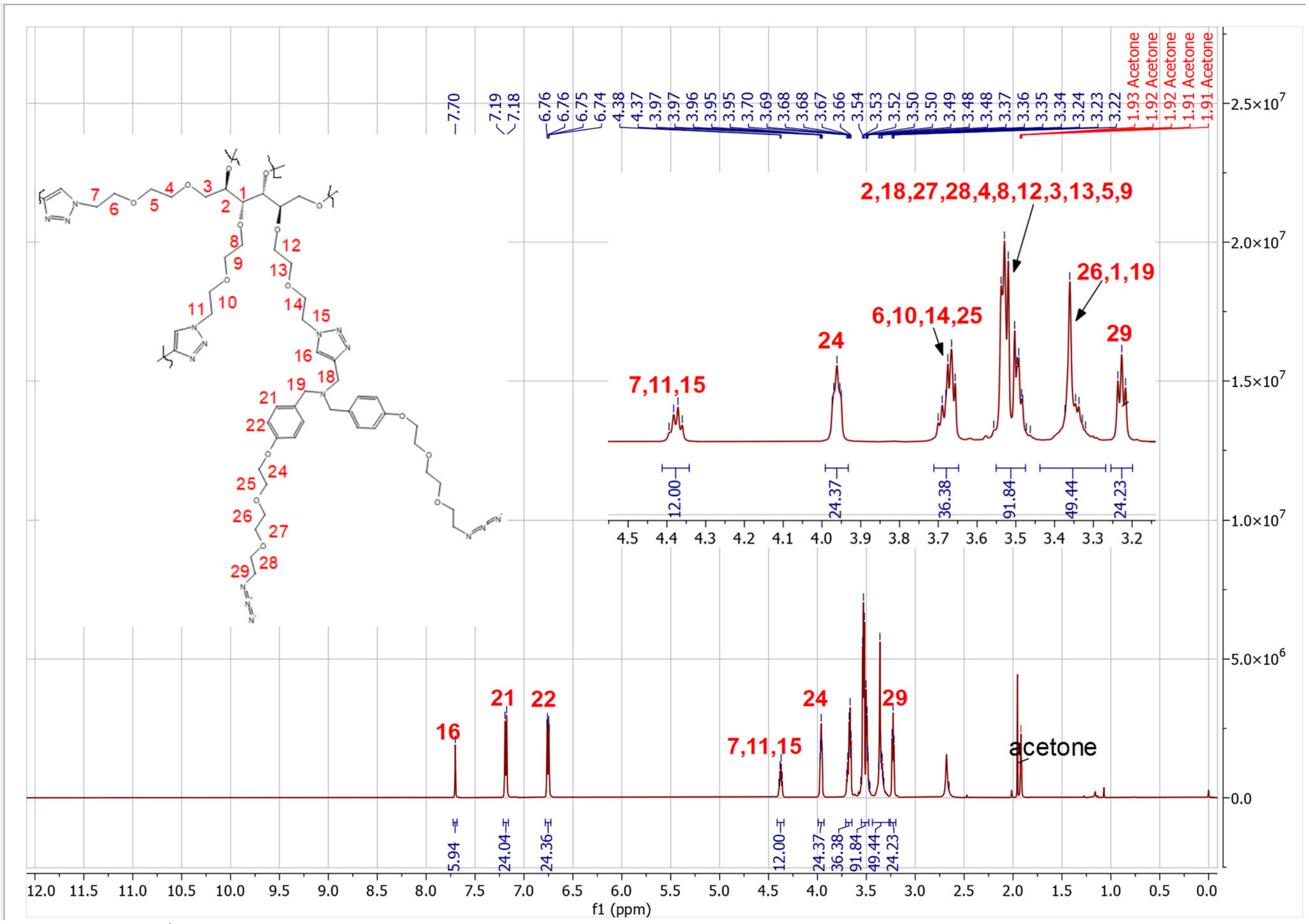

Figure S21. ^{1}H NMR spectrum of compound **7**.

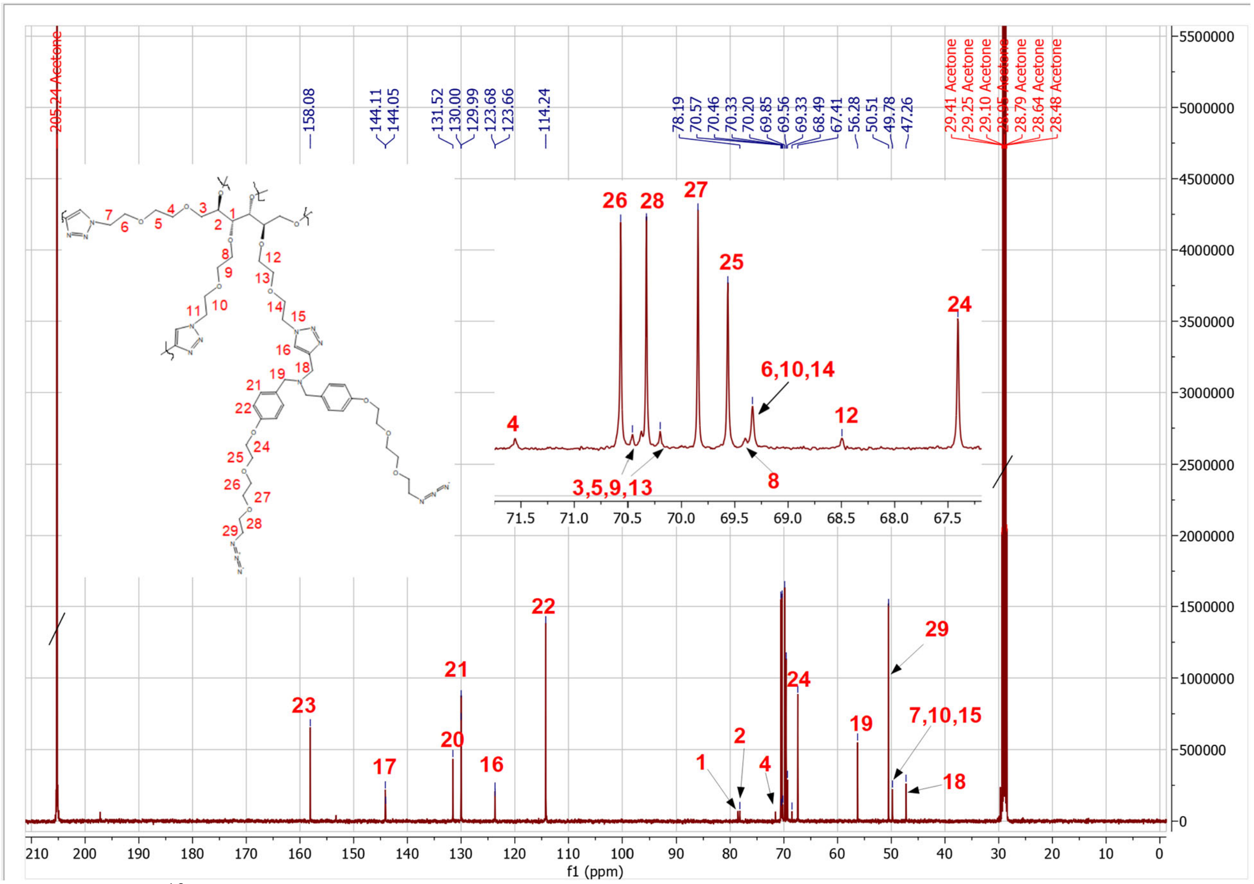

Figure S22. ^{13}C NMR spectrum of compound **7**.

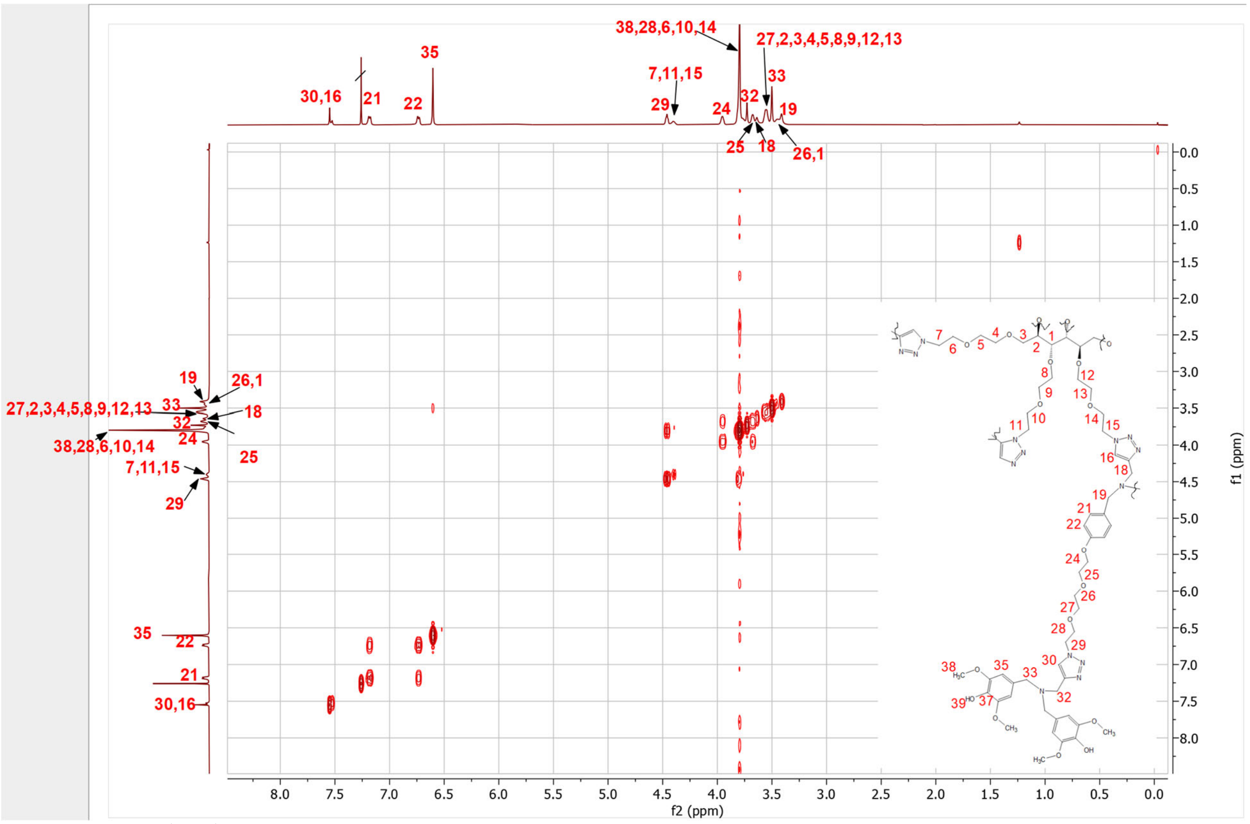

Figure S23. ^{1}H-^{1}H COSY spectrum of compound **8**.

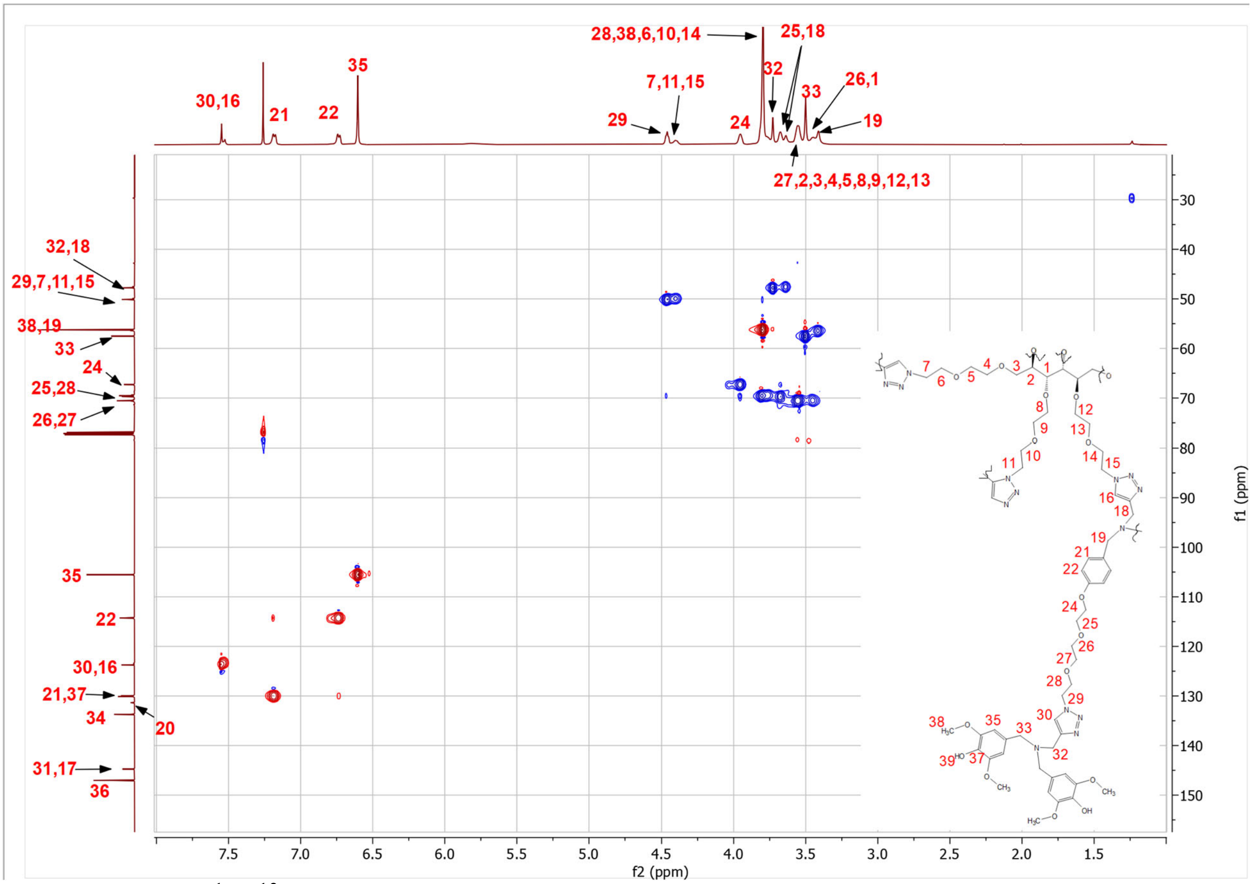

Figure S24. 2D ^{1}H-^{13}C HSQC spectrum of compound **8**.

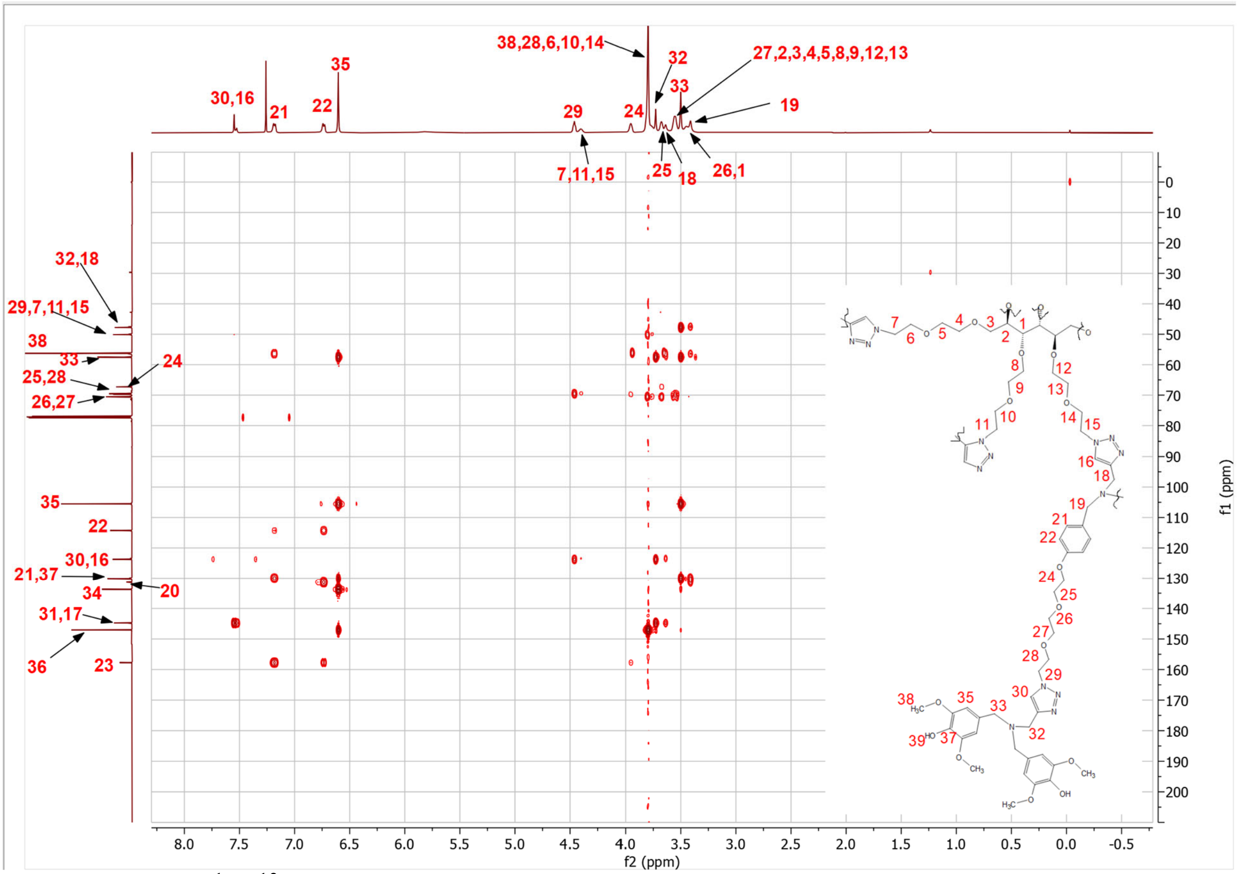

Figure S25. 2D ^{1}H-^{13}C HMBC spectrum of compound **8**.

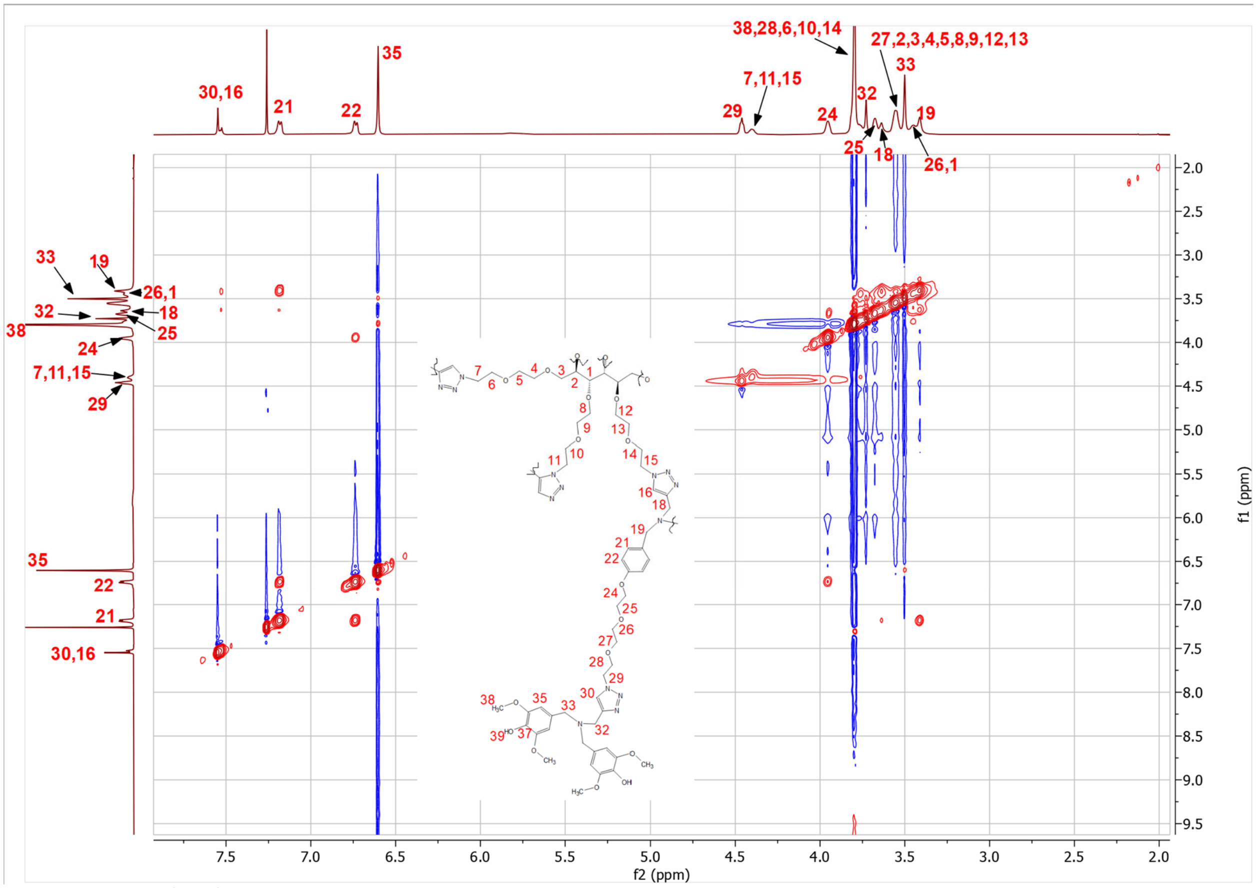

Figure S26. ^{1}H-^{1}H NOESY spectrum of compound **8**.
